# The Role of HbA1c Determination in Detecting Unknown Glucose Disturbances in Ischemic Stroke

**DOI:** 10.1371/journal.pone.0109960

**Published:** 2014-12-08

**Authors:** Jaume Roquer, Ana Rodríguez-Campello, Elisa Cuadrado-Godia, Eva Giralt-Steinhauer, Jordi Jiménez-Conde, Carol Soriano, Angel Ois

**Affiliations:** 1 Servei de Neurologia, IMIM-Hospital del Mar, DCEXS, Universitat Pompeu Fabra, Barcelona, Spain; 2 Servei de Neurologia, IMIM-Hospital del Mar, Department de Medicina, Universitat Autònoma de Barcelona, Barcelona, Spain; 3 Servei de Neurologia, IMIM-Hospital del Mar, Barcelona, Spain; University of Catanzaro Magna Graecia, Italy

## Abstract

**Objectives:**

To evaluate the usefulness of hemoglobin A1c (HbA1c) determinations during the acute ischemic stroke (IS) to identify undiagnosed glucose disturbances in a prospective series of patients with first-ever IS.

**Methods:**

Retrospective analysis of a prospective series of first-ever IS patients. Patients with previous diagnosis of diabetes mellitus (DM) were excluded from the study. Patients were classified as non-DM (HbA1c<5.7% and no previous evidence of 2 or more fasting blood glucose> = 126 mg/dL), prediabetes (HbA1c from 5.7% to 6.4%), and new suspected DM (HbA1c> = 6.5% independently of current blood glucose). Medical charts from hospital discharge to July 2014 of all suspected DM patients were reviewed to confirm the DM diagnosis.

**Results:**

The initial cohort included 1283 patients, of which 393 were excluded because of previous DM diagnosis and 136 because HbA1c during acute stroke phase was not available. No demographic differences were observed between patients with and without HbA1c determinations. The final cohort was composed of 754 patients with first-ever IS and unknown DM history. HbA1c determination suggested new DM in 87 cases (11.5%) and detected 273 patients with prediabetes (36.2%). New DM cases were identified in all etiological stroke subtypes. After discharge, DM diagnosis was confirmed in 80.2% of patients with available follow-up.

**Conclusions:**

HbA1c determination detected both undiagnosed DM and prediabetes in IS patients without taking into account the blood glucose values during admission, and independently of etiological stroke subtype. HbA1c determination should be included in the systematic screening of all IS patients.

## Introduction

Diabetes mellitus (DM) is a risk factor for stroke [1], [2] and is very common in acute ischemic stroke (IS) cases. The prevalence of DM in acute IS ranges from 15% to 44% [3]–[7]; the actual frequency depends on the criteria and methodology used during hospitalization to identify DM; currently, hemoglobin A1c (HbA1c) screening is probably the best single test [8]. In-hospital DM diagnosis is generally made in patients with a verified DM history, but an additional 6% to 42% of hospital-diagnosed DM was unrecognized before the patient's stroke [9], [10]. On the other hand, unknown DM in acute IS could be over estimated by the high prevalence of poststroke hyperglycemia: approximately 40% of patients with acute IS have admission blood glucose> = 130 mg/dL [11]. Although it is believed that the majority of such patients have DM or prediabetes, the possibility of poststroke-only hyperglycemia is also possible in some cases [12]. Since 2010, the American Diabetes Association guidelines [13] have included HbA1c as a method to diagnose DM (HbA1c levels> = 6.5%) and prediabetes (HbA1c levels from 5.7% to 6.4%), but the impact of this recommendation at the time of hospitalization for stroke is unknown.

In the present study, we analyzed the prevalence of unrecognized DM according to HbA1c in patients with acute IS. Additionally, we assessed the relationship between first blood glucose (from emergency room records) and the presence of previously undiagnosed DM. Our hypothesis was that the determination of HbA1c during the acute phase of stroke increases the diagnoses of new DM cases in all stroke etiological subgroups. Additionally, we assessed the relationship between first blood glucose (at emergency room) and the presence of previously undiagnosed DM.

## Materials and Methods

From January 2007 to December 2012, 2137 patients with acute stroke were admitted to a single tertiary hospital. All patients were prospectively included in the BASICMAR database [14], an ongoing register of patients with acute stroke at our hospital. For this study, patients with intracerebral hemorrhage (n = 292), transient ischemic attack (n = 289), previous stroke (n = 201), and unusual cause of stroke (n = 72) were excluded. From the remaining patients (n = 1283), we excluded 393 cases because of previous DM diagnoses and 136 because HbA1c determinations were not available, due to early discharge (n = 44), early death (n = 33), or determination not ordered (n = 59). Demographic characteristics of patients with and without HbA1c determination were compared. The final cohort was composed of 754 patients with first-ever IS and no previous history of DM.

In all patients, stroke severity was measured by the National Institutes of Health Stroke Scale (NIHSS) [15] by a trained neurologist upon hospital admission, and all received a computed tomography (CT) scan in the emergency room. Stroke subtype was categorized using the TOAST classification [16], following a neurovascular study that included carotid and transcranial ultrasound or angio-magnetic resonance imaging (MRI), and 24-hour electrocardiogram (EKG) monitoring. Additional CT or MRI evaluations were done, if needed, during hospitalization. Transthoracic or transesophageal echocardiography was performed in patients with strokes of undetermined origin. Treatment followed national and international guidelines, and included rtPA treatment (first 4.5 hours) and, beginning in 2010, endovascular treatment. The first blood glucose (nonfasting) was obtained in the emergency room, and is the value used for this study. During hospitalization, additional glucose testing was done when indicated by systematic protocol and the patient's clinical progress. HbA1c determination was obtained from fasting patients on the morning after admission or during the first 7 days of hospitalization.

### Factors analyzed

Vascular risk factors, as defined by international guidelines, were obtained from the patient, relatives, caregivers, or previous medical records. A structured questionnaire was used to record the following: arterial hypertension (evidence of at least two raised blood pressure measurements, systolic>140 mmHg or diastolic>90 mmHg, recorded on different days before stroke onset; a physician's diagnosis; or use of medication); diabetes (previous physician diagnosis or use of medication); hyperlipidemia (physician diagnosis, use of medication, serum cholesterol concentration>220 mg/dL, low density lipoprotein cholesterol [LDL-c]>130 mg/dL, or serum triglyceride concentration>150 mg/dL); current smoking habits; ischemic heart disease (documented history of angina pectoris or myocardial infarction), and atrial fibrillation (AF) (physician diagnosis, use of medication, or conclusive electrocardiogram data). Body mass index (BMI) and waist circumference (WC) were obtained in 669 and 591 patients, respectively.

**Glucose disturbances:.** Using the HbA1c value obtained during hospitalization and after reviewing medical charts for evidence of 2 or more fasting blood glucoses> = 126 mg/dL, patients were classified as follows: 1) Non-DM if HbA1c<5.7% and no previous evidence of hyperglycemia; 2) Prediabetic if HbA1c from 5.7 to 6.4% and no previous evidence of hyperglycemia; 3) New suspected DM if HbA1c> = 6.5% independently of current blood glucose. Medical charts of all patients with suspected new DM during admission were reviewed from the hospital discharge date to July 2014 to identify those cases in which the final DM diagnosis was confirmed.

### Statistical analysis

Age, NIHSS, blood glucose at emergency room, BMI, WC, and HbA1c presented a non-normal distribution and were expressed as medians and interquartile ranges 25–75(IQR 25–75). Categorical data were expressed as real numbers and percentages. Differences in parametric and nonparametric continuous variables were evaluated using the t test and Mann–Whitney U test/Kruskal-Wallis test, respectively, and the chi square test was used for proportional analysis.

We analyzed the relationship between final diagnoses of glucose disturbance (non-DM, prediabetes, and DM) and the TOAST subtypes (atherothrombotic, cardioembolic, lacunar, and undetermined). Finally, using the Kruskal–Wallis test to analyze the relationship between first blood glucose and the diagnosis of DM or prediabetes, patients were categorized into four groups according to their initial blood glucose: <127 mg/dL, 127 to 150 mg/dL, 151–200 mg/dL, and> = 200 mg/dL). All analyses were two-tailed. The significance level was set at 0.05.

### Ethics

The information used in this study was collected from the prospective BASICMAR register. The register, and its use for clinical or epidemiological studies, was approved by the IMIM-Hospital del Mar ethics committee. All patients gave their written informed consent prior to their inclusion in the study. Patient identifiers were removed prior to analysis.

## Results

Vascular risk factors, stroke characteristics, and demographic and outcome data of patients with (n = 1088) and without (n = 195) HbA1c determination are shown in Table 1. There were no differences between both groups in age, sex, previous DM diagnoses (30.7% vs 30.3%), glucose at admission (121 mg/dl vs 129 mg/dl), vascular risk factors, stroke severity by NIHSS (5 vs 6), BMI, and WC. Mortality was higher in patients without HbA1c determination (7.6% vs 22.6%, p<0.0001).

**Table 1:** Demographic and baseline data of patients with first-ever ischemic stroke and HbA1c determination (n = 1088), and those without HbA1c determination (n = 195).

	Total cases	With HbA1c	Without HbA1c	p, OR (95%CI)
	(n = 1283)	(n = 1088)	(n = 195)	
Age, median years, (IQR 25,75)	77 (68 83)	77 (67 83)	77 (69 85)	0.179
Men, n (%)	641 (50.0)	539 (49.5)	102(52.3)	0.485
Women, n (%)	642 (50.0)	549 (50.5)	93 (47.7)	
Initial DM diagnosis, n (%)	393 (30.6)	334 (30.7)	59 (30.3)	0.902
New suspected DM diagnosis, n (%)	92 (7.2)	87 (8.0)	5 (2.6)	0.004, 3.30 (1.32–8.24)
Blood glucose*, median mg/dL(IQR 25,75)	103 (122 159)	121 (102 157)	129 (105 167)	0.060
Arterial hypertension, n (%)	933 (73.0)	791 (73.1)	142 (72.8)	0.930
Dyslipidemia, n (%)	553 (43.3)	484 (44.6)	73 (38.0)	0.970
IHD, n (%)	196 (15.4)	164 (15.2)	32 (16.6)	0.590
PAD, n (%)	113 (8.9)	93 (8.6)	20 (10.3)	0.413
AF, n (%)	462 (36.0)	394 (36.2)	68 (34.9)	0.746
Current smoking, n (%)	258 (20.5)	222 (20.7)	36 (19.0)	0.627
Alcohol overuse, n (%)	234 (18.6)	207 (19.4)	27 (14.2)	0.015
NIHSS at admission, median (IQR 25,75)	5 (3 13)	5 (3 12)	6 (2 16)	0.270
BMI men, median (IQR 25,75)	26.5(24.1 29.3)	26.6 (24.2 29.4)	25.9 (24.5 28.3)	0.190
BMI women, median (IQR 25,75)	27.1(24.0 30.8)	27.2 (24.2 31.1)	26 (22.2 305)	0.067
WC men, median (IQR 25,75)	100 (90 107)	100 (90 108)	100 (90 104.5)	0.770
WC women, median (IQR 25,75)	98 (89 106)	99 (89 107)	97.5 (87.7 105)	0.291
In-hospital mortality, n (%)	127 (9.9)	83 (7.6)	44 (22.6)	0.0001

* Blood glucose (not fasting) obtained at emergency room.

DM = diabetes mellitus; IHD = ischemic heart disease; PAD = peripheral arterial disease;

AF = atrial fibrillation; BMI = body mass index; WC = waist circumference.

Mean baseline age of the 754 patients with first-ever IS and no previous history of DM was 77 years; 48.1% were men. Past history of arterial hypertension was present in 68.9% of cases, ischemic heart disease in 13.2%, peripheral arterial disease in 6.8%, hyperlipidemia in 39.4%, and atrial fibrillation in 37.1%. The rest of the baseline data are shown in Table 2.

**Table 2:** Demographic and baseline data of all patients with first-ever ischemic stroke included in the study (n = 754) after exclusion of patients with previous DM and with no HbA1c determination.

Variable	Final cohort
	(n = 754)
Age, median years, (IQR 25,75)	77 (67 83)
Men, n (%)	363 (48.1)
Women, n (%)	391 (51.9)
Blood glucose*, median mg/dL (IQR 25,75)	113 (99 133)
Diabetes mellitus, n (%)	-
Hypertension, n (%)	517 (68.9)
Ischemic heart disease, n (%)	99 (13.2)
Peripheral arterial disease, n (%)	51 (6.8)
Hyperlipidemia, n (%)	296 (39.4)
BMI men, median (IQR 25,75)	26.0 (23.7 29.0)
BMI women, median (IQR 25,75)	26.7(24.2 30.2)
WC men, median (IQR 25,75)	99 (90 106)
WC women, median (IQR 25,75)	98 (88 106)
Atrial fibrillation, n (%)	280 (37.1)
Current smoking, n (%)	163 (22.0)
Current alcohol overuse, n (%)	142 (19.2)
Atherothrombotic stroke, n (%)	94 (12.5)
Lacunar stroke, n (%)	168 (22.3)
Cardioembolic stroke, n (%)	293 (38.9)
Undetermined stroke, n (%)	199 (26.4)

* Blood glucose (not fasting) obtained at emergency room.

BMI = body mass index; WC = waist circumference.

During hospitalization, HbA1c determination revealed 87 new suspected DM cases (11.5%). In 25 cases, HbA1c suggested the DM diagnosis despite normal blood glucose at admission (<127 mg/dL). Prediabetes was observed in 273 patients (36.2%), and only 394 (52.3%) had no glucose disturbances. In patients with suspected new DM, BMI (IQR) was non-significantly higher than in non-DM patients (27.6 [24.3, 30.1] vs 26.2 [23.8, 29.1], p = 0.053), and WC was significantly higher (101 [92, 109.8] vs 96 [87, 104], respectively, p = 0.005).

**Figure 1:**
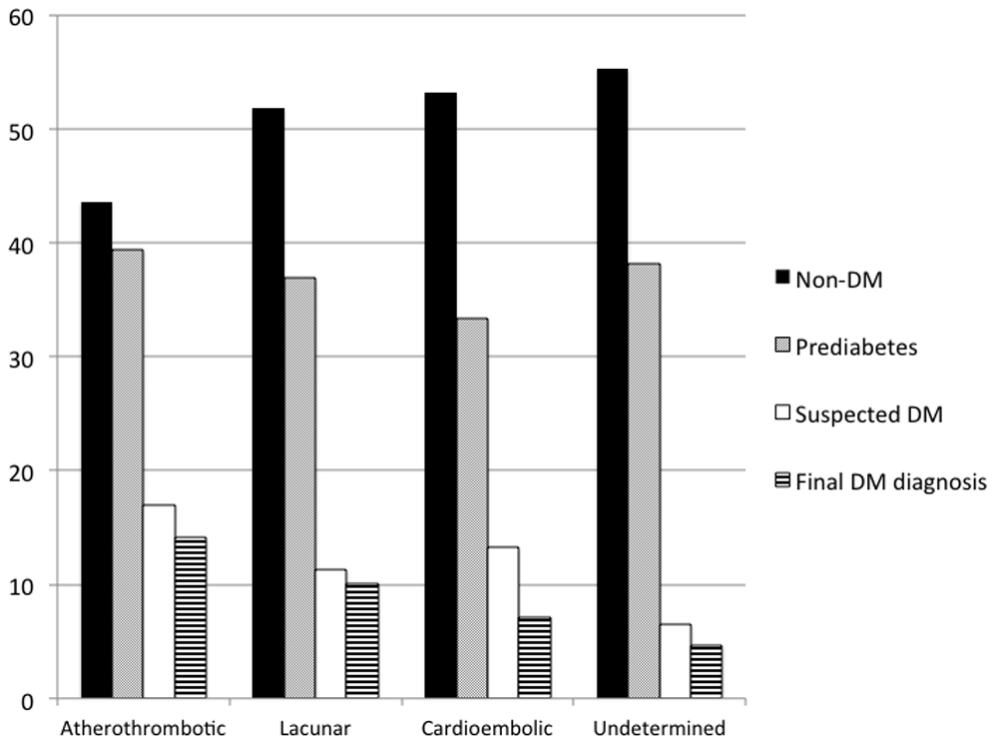
Glucose disturbance diagnoses during hospitalization (Non-DM, Prediabetes and Suspected DM), p = 0.107, and final DM diagnosis according to TOAST subtype, p = 0.072.

There were no significant differences between glucose disturbance groups in TOAST subtypes (Fig. 1): atherothrombotic strokes, 41 (43.6%) in non-DM group, 37 (39.4%) in prediabetics, and 16 (17.0%) in suspected diabetics; lacunar strokes, 87 (51.8%) in non-DM group, 62 (36.9%) in prediabetics, and 19 (11.3%) in suspected diabetics; cardioembolic strokes, 156(53.2%) in non-DM group, 98 (33.4%) in prediabetics, and 39 (13.3%) in suspected diabetics; and undetermined strokes, 110 (55.3%) in non-DM group, 76 (38.2%) in prediabetics, and 13 (6.5%) in suspected diabetics (p = 0.107). The figures for confirmed DM after follow-up were 13 (14.1%) atherothrombotic, 17 (10.1%) lacunar, 20 (7.1%) cardioembolic, and 9 (4.6%) undetermined strokes, p = 0.072.

There were non-significant (p = 0.110) differences between TOAST groups in HbA1c values: for atherothrombotic strokes, 5.7 (5.3, 6.2); lacunar strokes, 5.5 (5.2, 5.9); cardioembolic strokes, 5.5 (5.1, 6.0), and undetermined strokes, 5.5 (5.1, 5.9).

**Figure 2:**
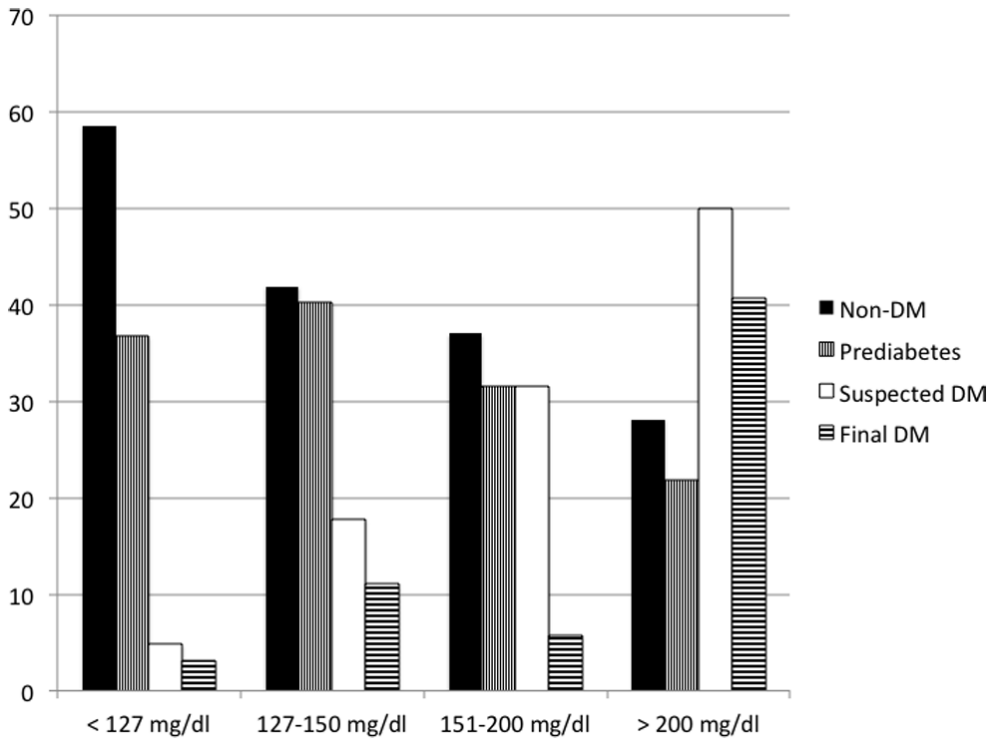
Glucose disturbance diagnosis during hospitalization (Non-DM, Prediabetes and Suspected DM in percentages), and Final DM diagnosis according to the emergency room glucose level (not fasting) in patients with acute IS, no previous diagnosis of DM, and HbA1c determination. p<0.0001.

The analysis of the relationship between first blood glucose determination and final suspected DM/prediabetes diagnoses showed that diagnosis of DM significantly increases (p<0.0001) with higher first blood glucose values: (4.8%) of patients with no past history of DM and normal blood glucose at admission (<127 mg/dL), 23 (17.8%) with glucose between 127 and 150 mg/dL, 23 (31.5%) with glucose between 151–200 mg/dL, and 16 (50.0%) of those with a value> = 200 mg/dL were finally diagnosed as suspected DM (Fig. 2). For prediabetes, the results were the following: 191 (36.7%) patients with normal blood glucose at admission, 52 (40.3%) with glucose between 127 and 150 mg/dL, 23 (31.5%) with glucose between 151–200 mg/dL, and 7 (21.9%) of those with a value> = 200 mg/dL in first blood glucose were finally diagnosed as prediabetics. A final non-DM diagnosis was observed in, 304 (58.5%) patients with normal blood glucose at admission, 54 (41.9%) with glucose between 127 and 150 mg/dL, 27 (37.0%) with glucose between 151–200 mg/dL, and 9 (28.1%) of those with a value> = 200 mg/dL.

After reviewing the medical charts of the 87 patients with suspected DM, in 14 cases it was not possible to confirm the DM diagnosis because of early death (n = 11), no data available (n = 2), or no new Hb1Ac determinations ordered during the follow-up (n = 1). Among the 73 patients with follow-up, DM was confirmed in 59 cases (80.8%) and ruled out in 14. When DM was ruled out, the glucose values ranged from 90 to 196 mg/dL, and the HbA1c from 6.5% to 7.0%.

## Discussion

Current DM diagnostic criteria [17] include a fasting plasma glucose level> = 126 mg/dL (7.0 mmol/L), or HbA1c> = 6.5%, or a casual plasma glucose> = 200 mg/dL (11.1 mmol/L) in the setting of symptoms attributable to hyperglycemia. In patients with acute stroke and acute hyperglycemia without known DM, it can be difficult to ensure a new DM diagnosis [18]. In this situation, HbA1c determination permits the exclusion of nondiabetic hyperglycemia as well as the identification of unknown DM patients admitted with normal glucose levels [12]. Regarding prediabetes diagnosis, the situation is quite similar: a plasma glucose range from 100 to 126 mg/dL is very common in the acute stroke phase, but does not by itself permit the diagnosis of prediabetes [18]. Moreover, a vast majority of patients are unaware of whether or not they have prediabetes [19].

The value of HbA1c determination during acute IS has been previously evaluated in two small series of patients [20], [21] and in 700 cases of stroke that included IS, transient ischemic attack (TIA), and intracerebral hemorrhage [22]. To our knowledge, our study is the largest to analyze HbA1c in patients with first-ever IS. Moreover, our patients were prospectively included in a structured database of all stroke cases admitted to a tertiary stroke center, and 84.7% of the patients in the series received HbA1c determination during admission. In addition, because no relevant differences were observed between patients with or without HbA1c determinations (Table 1), our data can be considered representative of the blood glucose disturbances present in first-ever acute IS.

Our study shows a 30.7% prevalence of known DM before stroke, very close to a previous report (30%) in our country [23] and within the range previously reported in Europe and North America [3]–[7]. This prevalence increases to 38.7% as a consequence of HbA1c determination during admission. In other words, in 11.5% (87 cases) of patients with acute stroke with no previous DM diagnosis, a new DM diagnosis was suspected, representing 20.7% of the final DM diagnoses. This figure might overestimate the real DM frequency, because the diagnosis was confirmed after discharge in only 80.2% of suspected DM cases for which follow-up HaA1c determinations were available. Two studies [20], [21] have analyzed the impact of HbA1c determination, in 107 and 166 non-DM patients with acute IS, respectively, and detected new DM diagnoses in 24% and 15% of cases, respectively. A very recent study [23], based on combined analysis of fasting plasma glucose, oral glucose tolerance test (OGTT), and HbA1c levels in patients with IS, TIA and intracerebral hemorrhage, reported that 27% of the patients had newly diagnosed DM and 52% were prediabetic. Previous studies [24]–[26] using the OGTT reported similar results (Table 3). No single screening test is fully adequate to screen for glucose disturbances in patients with acute IS [8]. It has been reported that the HbA1c cut-point of 6.5% identifies one-third fewer cases of undiagnosed DM than a fasting glucose cut-point of 126 mg/dL [27], and many studies have confirmed that OGTT screening diagnoses more people with DM than the HbA1c alone [28]. However, the HbA1c is considered the best single test [8]. According to the ADA recommendations, the HbA1c has several advantages over the OGTT, including greater convenience, greater preanalytical stability, and fewer day-to-day perturbations during periods of stress and illness [17]. In our study, it is interesting to note that HbA1c was useful to diagnose DM not only in patients with acute hyperglycemia, but also in those with normal glucose levels during acute stroke in whom there was no DM suspicion: 15 patients with <126 mg/dL glucose at admission had a confirmed DM, representing 26.8% of new DM diagnoses. In addition, HbA1c determination was useful for detecting new DM cases in all TOAST stroke subtypes (Fig. 1), with frequencies ranging from 6.5% in strokes of undetermined cause to 17.0% in atherosclerotic strokes (p = 0.110).

**Table 3:** Glucose disturbances in patients with acute IS and no past history of DM.

Study	Method used to diagnose DM	Cases (n)	New DM (%)	Prediabetes (%)	Total glucose disturbances (%)
Matz et al, 2006 (24)	OGTT	190	16.3	23	39.3
Urabe et al, 2009 (25)	OGTT	113	24.8	34.5	59.3
Jia et al, 2012 (26)	OGTT	1793	22.8	23.9	46.7
Dave et al, 2010 (20)	HbA1c+OGTT	107	24.0	37.0	61.0
Huisa et al, 2013 (21)	HbA1c	166	15.0	53.0	68.0
Fonville et al, 2013 (22)*	HbA1c+OGTT	700	27.0	52.0	79.0
Present (†)	HbA1c	754	11.5**	36.2	47.7

OGTT = oral glucose tolerance test.

* Included 374 IS cases, 269 TIA cases, and 57 intracerebral hemorrhage cases.

† First-ever ischemic strokes.

** This percentage refers to in-hospital suspected DM diagnoses. Final DM diagnosis was confirmed in 8.0% of cases.

Prediabetes is an independent risk factor for stroke [29], [30], but most patients with acute stroke are not aware of whether or not they are prediabetic [19]. HbA1c determination is currently accepted for prediabetes diagnoses [17]; therefore, testing it during IS hospitalization would be a good strategy to improve health after IS. Previous data, from both OGTT and HbA1c analysis during acute stroke, have shown a high prevalence of prediabetes in IS patients (Table 3), ranging from 23% to 53%; our results (36.2%) fall squarely in the middle of this range. Furthermore, prediabetic diagnoses were distributed across the four TOAST etiological groups in our cohort (Fig. 1).

Hyperglycemia is frequent in patients with acute cerebrovascular disease, whether diabetic or nondiabetic [12]. In patients with no past history of diabetes, often it is unknown whether the hyperglycemia is transient and due to the acute stress response or represents undiagnosed abnormal glucose metabolism [18]. In our study, blood glucose> = 126 mg/dL at admission was observed in 31.0% of patients with no past history of DM. The relationship between first glucose determination and final DM diagnoses was statistically significant (p<0.001). According to our data, the prevalence of suspected DM in IS patients with unknown DM and initial elevated nonfasting glucose is high: 31.5% for glucose from 151 to 200 mg/dL and 50.0% for glucose> = 200 mg/dL. In other words, the percentage of stress hyperglycemia is around 50% in patients with acute stroke and unknown DM with initial blood glucose greater than 200 mg/dL.

### Limitations of the study

Despite normality of glucose levels and HbA1c determinations during admission, a proportion of patients are likely to have DM or prediabetes. On the other hand, the ADA has recommended repeating HbA1c to rule out laboratory error [17]. In our cohort, the diagnosis of DM was confirmed in 67.8% of cases (n = 59), and was ruled out in 14 during thefollow-up. New HbA1c determinations were not available in 14 patients (11 due to early death, 2 foreign patients lost to follow-up, 1 not ordered). Among the 73 patients with follow-up, DM was confirmed in 80.8% of cases).

In conclusion, HbA1c determination provides relevant information in IS patients without known DM. It increases the detection of new DM cases, identifies prediabetic patients, and helps to differentiate the subgroup of patients with acute stress hyperglycemia. HbA1cdetermination should be obtained in all patients with first-ever IS without taking into account their blood glucose values during admission, and independently of etiological stroke subtype.

## Supporting Information

S1 HbA1c Data File containing clinical data of all cases of the study.
(PDF)
