# Supplementary material for: The Role of HbA1c Determination in Detecting Unknown Glucose Disturbances in Ischemic Stroke
Source: PLoS One. 2014 Dec 8;9(12):e109960. doi: 10.1371/journal.pone.0109960 (PMC4259295; doi:10.1371/journal.pone.0109960)
Supplement: S1 HbA1c Data — File containing clinical data of all cases of the study. (PDF) [file pone.0109960.s001.pdf]

Age	Sex	DM_Ver	DM_PDM	Hbglic	BMI	WC	diab_final	debutdm	glicini	nihini	e_TOAST_m	HT	Dyslip	PAD	CAD	FA	Enol_Cur	Sm_Curr	eExitus	iExitus	rankhist
75	1	0,00	0	4,9	22,46	79	0	0	107	5	1	0	0	0	0	0	0	1	1	1	0
74	0	0,00	0	5,1			0	0	85	13	1	1	1	0	0	0	0	1	1	1	0
86	1	0,00	0	5,6	25,95	98	0	0	209	21	1	0	0	0	0	0	0	0	1	1	1
77	1	0,00	0	5,3	25,83	90	0	0	125	21	1	1	1	0	0	0	1	0	1	1	0
77	1	1,00	1	6,0	24,39	105	0	0	110	3	1	1	1	1	0	0	1	0	1	1	0
69	1	3,00	2	7,1	23,12	80	1	1	257	19	1	1	1	0	1	0	1	0	1	1	1
83	0	0,00	0	5,1	16,41	75	0	0	156	19	3	0	1	0	0	0	0	0	1	1	1
87	0	0,00	0	4,7	26,16	72	0	0	89	18	3	0	0	0	0	1	0	0	1	1	2
94	0	0,00	0	5,1	20,81	90	0	0	94	23	3	1	0	0	0	1	0	0	1	1	3
81	0	0,00	0	5,1	29,14	103	0	0	276	23	3	1	0	0	0	1	0	0	1	1	0
85	1	0,00	0	5,1	22,86	84	0	0	91	21	3	1	0	0	0	0	1	0	1	1	0
94	1	0,00	0	5,3	28,34	105	0	0	116	17	3		0	0	0	1	0	0	1	1	3
87	1	0,00	0	4,6	25,10	82	0	0	97	16	3	1	1	0	1	0	0	0	1	1	3
93	0	0,00	0	4,6	20,81	80	0	0	114	20	3	1	0	0	0	1	0	0	1	1	0
81	0	0,00	0	5,5	24,84	109	0	0	208	15	3	1	0	0	1	1	0	0	1	1	0
91	0	0,00	0	5,1	28,93	95	0	0	183	16	3	1	0	0	0	1	0	0	1	1	0
69	0	0,00	0	4,7	31,25	104	0	0	119	24	3	0	0	0	0	1	0	0	1	1	1
71	0	0,00	0	5,5			0	0	166	19	3	1	1	0	0	1			1	1	0
82	1	0,00	0	5,3	27,68		0	0	128	21	3	1	0	0	0	1	0	0	1	1	0
99	0	0,00	0	5,0	35,56	95	0	0	126	20	3	1	1	0	0	1	0	0	1	1	1
86	0	0,00	0	5,4	28,40	103	0	0	116	19	3	1	0	0	0	1	0	0	1	1	0
85	0	0,00	0	5,0			0	0	144	31	3	1	0	0		1	0		1	1	0
85	0	0,00	0	5,6	22,04		0	0	122	21	3	1	1	0		1	0	0	1	1	3
92	0	0,00	0	5,6	27,06		0	0	125	17	3	0	1	0	1	1	0	0	1	1	3
83	1	0,00	0	4,7	21,67		0	0	104	20	3	1	0	0	0	1	0	0	1	1	2
83	1	0,00	0	5,2	53,58	97	0	0	114	12	3	0	0	0	0	1	0	0	1	1	0
90	0	0,00	0	5,3	23,14	80	0	0	127	21	3	1	0	0	0	1	0	0	1	1	1
85	0	0,00	0	5,6	22,22	92	0	0	109	18	3	1	0	0	0	1	0	0	1	1	2
79	1	0,00	0	5,1	22,53	90	0	0	159	23	3	1	0	0	0	1	0	0	1	1	2
89	0	0,00	0	5,3	23,44	105	0	0	104	10	3	1	1	0	0	1	0	0	1	1	0
69	1	0,00	0	5,5	27,68	92	0	0	120	21	3	1	1	0	0	1	0	0	1	1	1
79	0	0,00	0	5,3	35,56	73	0	0	137	17	3	1	0	0	0	1	0	0	1	1	0
64	0	1,00	1	5,9	42,97	120	0	0	109	23	3	1	0	0	0	1	0	0	1	1	0
88	1	1,00	1	5,7			0	0	144	16	3	1	0	0	0	1	0	0	1	1	0
79	1	1,00	1	6,3	23,15		0	0	126	17	3	0	0	0	1	1	1	0	1	1	0
92	0	1,00	1	6,2			0	0	187	23	3	1	0	0	0	1	0		1	1	2

87	0	1,00	1	6,0	26,67	94	0	0	102	20	3	1	0	0	0	1	0	0	1	1	3
90	0	1,00	1	6,2	33,79	104	0	0	108	9	3	1	1	0	0	1	0	0	1	1	3
75	0	1,00	1	6,2	27,64	87	0	0	108	14	3	1	0	0	1	1	0	0	1	1	0
87	0	1,00	1	6,4			0	0	223	20	3	1	0	0	0	1	0	0	1	1	0
71	0	3,00	2	8,3	27,55	105	1	1	251	16	3	1	1	0	0	1	0	0	1	1	1
83	0	3,00	2	8,3	27,66	92	1	1	177	19	3	1	1	0	0	1	0	0	1	1	2
94	0	3,00	2	6,7	23,61	75	1	1	215	25	3	0	0	0	1	1	0	0	1	1	2
90	0	3,00	2	6,5	26,67		1	1	195	22	3	0	0	1	1	1	0	0	1	1	3
82	0	3,00	2	6,5	27,34		1	1	218	22	3	1	0	0	0	1	0	0	1	1	0
73	1	3,00	2	6,7			1	1	121	23	3	0	0	0	0	1	1		1	1	0
88	0	2,00	2	7,0	29,38	110	1	1	159	21	3	1	0	0	0	1	0	0	1	1	2
87	0	2,00	2	7,6	25,81	80	1	1	115	23	3	1	1	0	0	1	0	0	1	1	0
81	0	0,00	0	5,2	36,44	135	0	0	107	16	4		0	0	0	0	0	0	1	1	0
85	0	0,00	0	5,2	35,56	90	0	0	132	19	4	0	0	0	0	0	0	0	1	1	2
86	1	0,00	0	5,4	27,34	97	0	0	74	8	4	0	0	0	0	1	1	0	1	1	2
53	1	0,00	0	5,5	19,03	86	0	0	84	18	4	0	0	0	0	1	0	1	1	1	1
99	0	0,00	0	5,5			0	0	135	18	4	1	0	0	0	0	0	0	1	1	3
81	1	0,00	0	5,1	24,49	120	0	0	98	21	4	1	0	0	1	1	1	1	1	1	3
78	0	1,00	1	5,5	34,05	90	0	0	132	22	4	1	1	0	0	0	0	0	1	1	0
68	1	1,00	1	5,8	22,55		0	0	269	9	4	1	1	1	0	1	0	0	1	1	1
90	0	1,00	1	6,4	31,11	92	0	0	111	18	4	1	1	0	0	0	0	0	1	1	3
63	1	0,00	0	5,3	25,10	95	0	0	139	22	1	0	0	1	0	0	0	1	1	0	0
72	1	1,00	1	6,0	32,65	110	0	0	168	24	1	1	1	0	0	0	1	0	1	0	0
98	0	0,00	0	5,1	25,11	89	0	0	110	17	2	1	1	0	0	1	0	0	1	0	3
95	0	0,00	0	4,6	21,33	78	0	0	111	11	3	1	0	0	0	1	0	0	1	0	0
76	0	0,00	0	4,6	24,61	97	0	0	129	17	3	1	1	0	0	1	0	0	1	0	0
88	0	0,00	0	4,9	26,30	111	0	0	106	14	3	0	0	0	0	1	0	0	1	0	1
81	0	0,00	0	5,0	29,30	114	0	0	106	16	3	1	0	0	0	1	0	0	1	0	0
91	0	0,00	0	4,0	28,30	85	0	0	144	17	3	1	0	0	0	1	0	0	1	0	0
83	1	0,00	0	4,1	28,71	83	0	0	92	14	3	0	0	1	0	1	0	0	1	0	0
91	1	0,00	0	5,1	23,53	78	0	0	157	17	3	1	0	0	0	1	0	0	1	0	1
98	0	0,00	0	4,9	27,24	90	0	0	103	8	3	0	0	0	0	1	0	0	1	0	3
81	1	0,00	0	5,3	23,31	127	0	0	110	18	3	0	0	0	0	1	0	0	1	0	2
71	1	0,00	0	5,5	26,87		0	0	107	18	3	1	0	0	0	1	0	0	1	0	0
91	0	0,00	0	5,5			0	0	73	6	3	1	1	0	1	1	0	0	1	0	2
79	0	0,00	0	5,4	20,81	63	0	0	100	17	3	0	0	0	0	1	0	0	1	0	1
85	1	0,00	0	5,6	22,49	93	0	0	92	6	3	1	0	0	0	1	0	0	1	0	2

82	0	0,00	0	5,6	24,61	90	0	0	98	5	3	1	1	0	0	1	0	0	1	0	2
81	1	0,00	0	5,5	29,76	110	0	0	95	3	3	0	1	0	0	1	0	0	1	0	0
77	1	1,00	1	5,3	30,07		0	0	108	2	3	1	0	0	0	1	0	0	1	0	1
70	1	1,00	1	6,1			0	0	115	4	3	1	0	0	0	1	1	0	1	0	0
76	0	1,00	1	5,7	28,91	102	0	0	170	14	3	1	0	0	0	1	0	0	1	0	0
86	0	1,00	1	6,0	26,04	105	0	0	96	19	3	1	0	0	0	1	0	0	1	0	3
85	0	1,00	1	6,1	26,67	107	0	0	160	19	3	1	0	0	0	1	0	0	1	0	2
95	0	1,00	1	5,8	16,53	75	0	0	204	19	3	1	1	0	0	1	0	0	1	0	3
68	1	1,00	1	6,0	24,22	106	0	0	86	19	3	1	0	0	0	1	0	0	1	0	3
89	0	1,00	1	6,0	23,44		0	0	137	20	3	1	0	1	0	1	0	0	1	0	1
87	1	1,00	1	6,1	21,56	93	0	0	85	2	3	1	0	0	1	1	0	0	1	0	3
95	0	1,00	1	6,3	26,67	92	0	0	144	12	3	1	0	0	1	1	0	0	1	0	3
68	0	1,00	1	5,8	29,09	104	0	0	118	5	3	1	1	0	0	1	0	0	1	0	0
99	0	1,00	1	5,7	22,04		0	0	87	22	3	1	1	0	0	1	0	0	1	0	3
86	0	1,00	1	6,1	33,33	110	0	0	107	4	3	1	0	0	0	0	0	0	1	0	3
88	0	3,00	2	7,0	26,30	101	1	1	100	23	3	1	1	0	0	1	0	0	1	0	0
77	0	0,00	0	5,5	31,25	108	0	0	112	4	4	1	1	0	0	1	0	0	1	0	1
93	0	0,00	0	5,4	31,11	110	0	0	170	21	4	1	0	0	0	0	0	0	1	0	3
87	1	0,00	0	4,8			0	0	120	13	4	1	0	0	0	0	0	0	1	0	3
87	0	0,00	0	5,0			0	0	120	12	4	1	1	0	0	0	0	0	1	0	3
80	0	0,00	0	5,4	27,24		0	0	127	22	4	1	0	0	0	0	0	0	1	0	1
85	1	0,00	0	5,6	35,55	106	0	0	112	16	4	1	0	0	0	0	0	0	1	0	2
87	1	1,00	1	5,9			0	0	116	20	4	1	1	0	0	0	0		1	0	0
88	1	1,00	1	5,7	23,44	90	0	0	114	7	4	0	0	0	0	0	0	0	1	0	0
75	1	1,00	1	6,0	29,40	96	0	0	122	12	4	1	1	0	0	0	0	0	1	0	1
82	1	3,00	2	6,8	28,89	90	1	1	210	21	4	0	1	0		0	1	1	1	0	2
58	1	0,00	0	4,9	20,28	85	0	0	88	4	1	0	1	0	0	0	0	1	0	0	1
81	0	0,00	0	4,9	31,99	115	0	0	110	1	1	1	1	0	1	0	0	0	0	0	1
55	1	0,00	0	4,9	26,26	100	0	0	95	1	1	1	0	0	0	0	0	1	0	0	0
55	1	0,00	0	4,7	23,70	89	0	0	133	8	1	0	0	0	0	0	0	1	0	0	0
85	0	0,00	0	4,1	27,77	90	0	0	108	10	1	1	0	0	0	0	0	0	0	0	0
86	0	0,00	0	5,2	33,30	104	0	0	113	11	1	1	1	0	0	0	0	0	0	0	3
72	1	0,00	0	5,1	25,35	89	0	0	91	8	1	0	1	0	0	0	1	1	0	0	0
76	1	0,00	0	5,5	27,82	98	0	0	158	6	1	0	1	0	0	0	1	0	0	0	0
80	1	0,00	0	4,7	24,07	105	0	0	82	4	1	0	0	0	0	0	0	0	0	0	1
91	1	0,00	0	4,9	27,17	102	0	0	100	0	1	0	0	0	0	0	0	0	0	0	0
62	0	0,00	0	4,8	17,98	78	0	0	110	6	1	0	0	0	0	0	0	1	0	0	0

79	1	0,00	0	4,6	22,72		0	0	121	0	1	1	0	0	1	0	0	0	0	0	0
77	0	0,00	0	4,4	25,71	105	0	0	118	10	1	1	0	0	0	0	0	0	0	0	0
60	1	0,00	0	5,1	22,31	94	0	0	91	0	1	1	1	0	0	0	1	1	0	0	0
73	0	0,00	0	5,2	24,97	83	0	0	90	8	1	1	1	0	0	0	0	1	0	0	0
75	0	0,00	0	5,2	25,39	84	0	0	110	5	1	1	0	0	0	0	0	0	0	0	0
66	1	0,00	0	5,2			0	0	101	21	1	0	1	0	0	0	0	1	0	0	0
64	1	0,00	0	5,5	27,44		0	0	127	14	1	1	1	0	1	0	0	0	0	0	0
80	0	0,00	0	5,4	26,67	103	0	0	97	5	1	0	1	0	1	0	0	0	0	0	2
90	0	0,00	0	5,0			0	0	99	5	1	0	0	1	0	0	0	0	0	0	0
86	1	0,00	0	5,4	25,26	83	0	0	82	7	1	1	1	0	0	0	1	0	0	0	0
73	1	0,00	0	5,4	28,03	99	0	0	101	1	1	1	1	0	0	0	1	1	0	0	0
48	1	0,00	0	5,3	29,63	102	0	0	225	19	1	1	1	0	0	0	1	1	0	0	0
82	1	0,00	0	5,4	31,14	120	0	0	115	7	1	1	1	0	1	0	1	0	0	0	3
78	1	0,00	0	5,3	26,08		0	0	87	3	1	0	0	0	0	0	0	0	0	0	0
89	0	0,00	0	5,3	24,24	102	0	0	115	6	1	1	0	0	0	0	0	0	0	0	2
67	1	0,00	0	5,6	21,64	90	0	0	142	1	1	0	0	0	0	0	0	1	0	0	0
68	0	0,00	0	5,6	28,44	99	0	0	123	2	1	0	1	0	0	0	0	1	0	0	0
87	0	0,00	0	5,3	29,30	108	0	0	160	17	1	1	0	0	0	0	1	0	0	0	3
65	1	0,00	0	5,4	21,63	80	0	0	127	8	1	0	0	0	0	0	1	1	0	0	2
62	1	0,00	0	5,5	21,46		0	0	228	6	1	1	1	1	0	0	1	1	0	0	0
50	1	0,00	0	5,6	30,42	110	0	0	119	1	1	1	1	0	0	0	1	1	0	0	0
75	1	0,00	0	5,3	31,14	102	0	0	87	10	1	1	0	0	0	0	1	1	0	0	3
60	0	0,00	0	5,4	20,78	69	0	0	104	2	1	1	0	0	0	0	0	1	0	0	0
62	1	0,00	0	5,6	36,36	86	0	0	80	4	1	0	0	0	0	0	0	1	0	0	0
52	1	0,00	0	5,6	25,51	127	0	0	166	3	1	1	0	0	0	0	1	1	0	0	0
80	1	1,00	1	5,6	24,31	90	0	0	108	6	1	0	0	0	0	0	0	0	0	0	0
64	1	1,00	1	5,8	29,73	115	0	0	106	1	1	1	1	1	1	0	1	1	0	0	0
64	1	1,00	1	5,3	33,12	115	0	0	89	0	1	1	0	0	0	0	0	0	0	0	0
74	0	1,00	1	5,3	23,51	104	0	0	86	4	1	1	1	0	0	0	0	0	0	0	0
70	0	1,00	1	5,4	26,95	114	0	0	101	10	1	1	0	0	0	0	0	1	0	0	0
88	0	1,00	1	5,4	23,73	88	0	0	107	5	1	1	0	0	0	0	0	0	0	0	0
58	1	1,00	1	5,7	25,25	106	0	0	141	22	1	1	0	0	0	0	1	1	0	0	0
81	1	1,00	1	6,3			0	0	70	4	1	1	0	0	0	0	0	1	0	0	0
60	0	1,00	1	6,1	35,16	122	0	0	137	7	1	1	1	0	0	0	0	1	0	0	0
59	1	1,00	1	6,4	29,88	114	0	0	90	0	1	0	1	1	1	0	1	1	0	0	0
76	1	1,00	1	5,8	26,57	102	0	0	93	20	1	1	1	0	0	0	1	1	0	0	0
60	1	1,00	1	6,4	38,51	117	0	0	120	7	1	1	1	0	1	0	0	0	0	0	0

59	1	1,00	1	6,2	24,22		0	0	167	3	1	1	0	0	0	0	1	1	0	0	0
79	0	1,00	1	5,8	26,56	106	0	0	78	7	1	1	1	0	0	0	0	0	0	0	3
80	1	1,00	1	5,9	27,25	100	0	0	107	6	1	1	0	0	0	0	1	1	0	0	3
83	1	1,00	1	5,8	23,66	82	0	0	142	20	1	0	0	0	0	0	0	1	0	0	1
78	0	1,00	1	5,8	29,30	120	0	0	121	4	1	1	1	0	0	0	0	0	0	0	0
82	1	1,00	1	5,7	24,80	89	0	0	82	6	1	1	0	0	0	0	1	1	0	0	0
82	1	1,00	1	6,0	21,72	94	0	0	109	8	1	1	0	1	0	0	0	0	0	0	0
63	1	1,00	1	5,7	26,03	98	0	0	150	16	1	0	0	0	0	0	1	1	0	0	0
69	1	1,00	1	5,8	29,58	107	0	0	133	2	1	0	1	0	0	0	0	0	0	0	0
76	1	1,00	1	6,1	30,84	113	0	0	148	19	1	0	0	0	0	0	1	0	0	0	0
48	0	1,00	1	5,8	24,86	106	0	0	59	8	1	1	1	0	0	0	0	1	0	0	0
68	1	1,00	1	5,8	26,67	96	0	0	102	4	1	1	1	0	0	0	0	0	0	0	0
64	1	1,00	1	5,7	23,51	93	0	0	121	4	1	0	0	0	0	0	1	1	0	0	0
62	1	1,00	1	5,7	33,46	121	0	0	163	0	1	1	1	1	0	0	1	1	0	0	0
85	1	1,00	1	6,3			0	0	103	1	1	1	0	0	0	0	1	0	0	0	2
74	1	1,00	1	5,7			0	0	145	8	1	1	0	1	0	0	0	1	0	0	1
67	1	1,00	1	6,4	24,62	90	0	0	108	5	1	1	1	1	0	0		1	0	0	3
80	1	1,00	1	6,3	28,73	94	0	0	101	9	1		1		0	0		0	0	0	2
73	0	1,00	1	5,7		159	0	0	101	1	1	1	1	0	0	0	1	0	0	0	0
72	1	1,00	1	5,8	25,39	92	0	0	111	21	1	1	0	0	0	0	0	1	0	0	0
70	0	1,00	1	5,8	22,41	80	0	0	95	2	1	0	0	0	0	0	1	1	0	0	0
81	1	1,00	1	6,4	16,23		0	0	151	1	1	1	0	1	0	0	0	0	0	0	2
51	1	1,00	1	5,7	21,37		0	0	98	0	1	0	0	0	0	0	0	1	0	0	0
72	0	3,00	2	6,5		94	1	1	104	1	1	1	1	0	0	0	0	1	0	0	0
64	1	2,00	2	6,5			1	1	151	11	1	0	0	1	0	0	1	1	0	0	0
35	1	2,00	2	7,0	30,85	102	1	1	133	2	1	1	1	0	1	0	1	1	0	0	0
69	1	2,00	2	6,7	28,34	101	1	1	169	5	1	0	0	1	0	0	0	0	0	0	2
64	1	2,00	2	10,5	25,71	85	1	1	286	9	1	0			0	0		0	0	0	0
77	0	2,00	2	6,6	22,48	90	1	1	112	0	1	1	1	0	1	0	0	0	0	0	0
82	1	2,00	2	10,4			1	1	260	2	1	1	1	0	1	0	0	0	0	0	0
76	1	2,00	2	6,6	32,03	105	1	1	114	3	1	1	0	0	0	0	1	0	0	0	0
86	0	2,00	2	6,7	33,73	111	1	1	129	4	1	1	1	0	1	0	0	0	0	0	3
74	1	2,00	2	7,4	23,88	92	1	1	196	4	1	0	1	0	0	0	0	0	0	0	0
73	1	2,00	2	6,6	22,11	97	1	1	111	1	1	1	1	1	0	0	0	1	0	0	1
68	0	2,00	2	8,5		94	1	1	167	9	1	1	0	0	0	0	0	1	0	0	3
81	1	2,00	2	10,4	29,30	102	1	1	272	2	1	1	1	0	0	0	0	0	0	0	0
76	0	2,00	2	6,6	24,21		1	1	155	3	1	1	1	0	0	0	0	0	0	0	0

79	1	0,00	2	6,8	21,13	96	1	1	127	4	1	1	1	0	1	0	0	0	0	0	0
64	0	0,00	0	5,1	24,97	90	0	0	96	4	2	0	0	0	0	0	0	1	0	0	0
78	1	0,00	0	5,3	24,34	100	0	0	88	2	2	1	0	0	0	0	0	0	0	0	0
84	0	0,00	0	5,2	26,56	93	0	0	91	4	2	0	0	0	0	0	0	0	0	0	0
80	0	0,00	0	5,4	22,39	98	0	0	103	3	2	1	0	0	0	0	0	1	0	0	3
54	0	0,00	0	4,7	29,67	100	0	0	118	3	2	1	1	0	0	0	0	0	0	0	2
53	1	0,00	0	5,1	21,51	92	0	0	90	7	2	0	1	0	0	0	0	1	0	0	0
91	1	0,00	0	4,4	28,06	99	0	0	103	6	2	0	0	0	0	0	0	1	0	0	0
71	1	0,00	0	4,6	39,06	140	0	0	114	2	2	1	1	0	0	0	0	0	0	0	0
89	0	0,00	0	4,9	24,97	100	0	0	112	10	2	1	0	0	0	0	0	0	0	0	0
61	1	0,00	0	4,7	20,31	71	0	0	94	6	2	1	0	0	0	0	0	1	0	0	0
71	1	0,00	0	4,2	23,51	90	0	0	103	3	2	1	0	0	0	0	1	1	0	0	2
59	0	0,00	0	4,7	31,14	104	0	0	97	10	2	0	1	0	0	0	0	0	0	0	0
74	0	0,00	0	5,0	31,11	82	0	0	122	3	2	0	0	0	0	0	0	0	0	0	0
53	1	0,00	0	4,4	30,84	102	0	0	79	2	2	0	1	0	0	0	0	0	0	0	0
63	1	0,00	0	5,1	22,32	87	0	0	120	4	2	0	0	0	0	0	1	1	0	0	0
75	0	0,00	0	5,1	24,22	82	0	0	98	8	2	1	0	0	0	0	0	0	0	0	0
72	0	0,00	0	5,0	30,36	104	0	0	101	3	2	1	0	0	0	0	0	0	0	0	0
71	1	0,00	0	4,8	27,04	105	0	0	106	4	2	0	0	0	0	0	1	1	0	0	0
92	1	0,00	0	4,6	24,21	82	0	0	88	4	2	1	1	0	0	0	1	1	0	0	2
58	1	0,00	0	4,7	29,64	96	0	0	103	2	2	0	0	0	0	0	0	0	0	0	0
62	0	0,00	0	5,3	26,42	105	0	0	147	4	2	1	0	0	0	0	0	0	0	0	0
54	1	0,00	0	4,7	27,45	89	0	0	90	3	2	0	1	0	0	0	1	0	0	0	1
87	0	0,00	0	4,3	25,30	90	0	0	80	4	2	1	0	0	1	0	0	0	0	0	1
76	1	0,00	0	5,0	27,64	106	0	0	99	6	2	0	1	0	1	0	1	0	0	0	0
57	1	0,00	0	4,5	30,45	110	0	0	82	0	2	1	1	0	0	0	0	0	0	0	0
68	1	0,00	0	5,2	29,74	103	0	0	145	5	2	1	1	0	0	0	1	0	0	0	0
62	1	0,00	0	5,1	35,16	119	0	0	88	3	2	1	1	0	1	0	0	0	0	0	0
43	0	0,00	0	5,0	21,62	73	0	0	135	3	2	0	0	0	0	0	0	1	0	0	0
41	1	0,00	0	4,9	24,51	92	0	0	149	8	2	0	0	0	0	0	0	0	0	0	0
72	1	0,00	0	4,4	23,95	102	0	0	102	15	2	0	0	0	0	0	0	1	0	0	0
77	1	0,00	0	5,5	27,92	96	0	0	64	1	2	0	0	1	0	0	0	0	0	0	0
56	1	0,00	0	4,8	23,03	91	0	0	100	12	2	0	0	0	0	0	1	1	0	0	0
71	0	0,00	0	5,5	32,00	95	0	0	205	7	2	0	1	0	0	0	0	0	0	0	0
81	1	0,00	0	4,9	25,54	88	0	0	94	6	2	0	1	0	0	0	0	1	0	0	3
75	1	0,00	0	4,9	22,53	102	0	0	103	7	2	0	0	0	0	0	0	0	0	0	3
80	0	0,00	0	5,2	26,64	93	0	0	118	1	2	1	1	0	0	0	0	0	0	0	0

86	0	0,00	0	4,7	21,64	67	0	0	89	7	2	1	0	0	0	0	0	0	0	0	3
49	1	0,00	0	4,5			0	0	114	1	2	0	1	0	0	0	0	1	0	0	0
63	1	0,00	0	5,3	26,18	86	0	0	180	3	2	1	1	1	0	0	1	1	0	0	0
71	1	0,00	0	4,3	21,71	86	0	0	117	0	2	1	1	0	0	0	0	0	0	0	0
49	0	0,00	0	4,8	33,06	73	0	0	93	3	2	1	0	0	0	0	0	0	0	0	0
74	1	0,00	0	4,7	24,80	95	0	0	100	2	2	1	1	1	1	0	1	1	0	0	1
62	0	0,00	0	5,6	27,18	88	0	0	115	2	2	1	1	0	0	0	0	0	0	0	0
77	0	0,00	0	5,4	30,83	86	0	0	171	4	2	1	0	0	0	0	0	0	0	0	0
88	0	0,00	0	5,0	25,39	106	0	0	94	3	2	0	0	0	0	0	0	0	0	0	0
82	1	0,00	0	4,8	24,97	90	0	0	91	3	2	1	1	0	1	0	0	0	0	0	3
51	1	0,00	0	4,2	23,38	100	0	0	94	1	2	1	1	0	0	0	0	0	0	0	0
82	1	0,00	0	5,5	28,69	85	0	0	78	5	2	0	1	0	0	0	0	0	0	0	1
76	1	0,00	0	4,4	25,23	100	0	0	98	4	2	1	0	0	0	0	1	0	0	0	0
66	1	0,00	0	4,9	28,36	115	0	0	108	0	2	1	0	0	0	0	1	1	0	0	0
71	0	0,00	0	5,0	27,64	105	0	0	96	5	2	1	0	0	0	0	0	0	0	0	1
65	1	0,00	0	5,0	24,22	84	0	0	95	5	2	0	0	0	0	0	0	0	0	0	3
54	1	0,00	0	4,8			0	0	72	4	2	1	0	0	0	0	0	1	0	0	0
79	1	0,00	0	5,0	26,95	80	0	0	79	2	2	1	0	0	0	0	0	0	0	0	0
79	1	0,00	0	5,2	26,37	87	0	0	103	4	2	0	1	0	0	0	1	0	0	0	0
69	1	0,00	0	4,7	34,25		0	0	111	3	2	1	0	0	0	0	1	1	0	0	0
59	0	0,00	0	5,2	27,56	86	0	0	99	2	2	0	0	0	0	0	0	0	0	0	0
77	1	0,00	0	5,2	29,05		0	0	91	3	2	1	0	1	0	0	1	0	0	0	0
66	0	0,00	0	5,5	28,04		0	0	116	1	2	1	1	0	0	0	0	0	0	0	0
52	0	0,00	0	5,3			0	0	112	6	2	1	1	0	0	0	1	1	0	0	0
65	1	0,00	0	4,9	34,77	110	0	0	95	12	2	1	0	0	1	0	0	0	0	0	2
70	1	0,00	0	5,5	22,20	97	0	0	131	2	2	1	0	0	0	0	1	0	0	0	0
55	1	0,00	0	5,4	29,38		0	0	184	4	2	1	0	0	0	0	1	1	0	0	0
84	0	0,00	0	5,4	25,39	102	0	0	106	9	2	0	1	0	0	0	0	0	0	0	0
51	1	0,00	0	4,7			0	0	87	1	2	1	0	0	0	0		1	0	0	0
55	1	0,00	0	5,2	31,12	106	0	0	101	2	2	1	1	0	0	0	1	1	0	0	0
75	1	0,00	0	5,5	21,45		0	0	107	3	2	0	1	1	0	0	0	1	0	0	1
73	1	0,00	0	5,1	35,16	132	0	0	119	6	2	1	1	0	1	0	0	0	0	0	3
81	1	0,00	0	5,6	29,72	117	0	0	100	5	2	0	1	0	0	0	0	1	0	0	0
76	1	0,00	0	5,6	28,13	99	0	0	150	2	2	1	0	0	0	0	0	0	0	0	0
30	1	0,00	0	5,4	27,77	124	0	0	100	6	2	0	1	0	0	0	0	0	0	0	0
75	1	0,00	0	5,4	31,22		0	0	82	6	2	1	0	0	0	0	1	1	0	0	2
58	0	0,00	0	5,1	24,89	85	0	0	93	7	2	1	0	0	0	0	0	0	0	0	2

64	1	0,00	0	5,6	23,85		0	0	103	7	2	1	1	0	0	0	0	0	0	0	0
63	0	0,00	0	5,6	36,03		0	0	96	3	2	1	1	1	0	0	0	0	0	0	1
73	0	0,00	0	5,6	30,80	111	0	0	104	5	2	1	1	0	0	0	0	0	0	0	0
55	1	0,00	0	5,4	24,80		0	0	98	4	2	0	0	0	0	0	0	1	0	0	0
81	1	0,00	0	5,5	26,23	115	0	0	94	1	2	1	1	0	0	0	1	0	0	0	2
66	0	0,00	0	5,5	27,83		0	0	90	12	2	1	0	0	0	0	0	0	0	0	3
79	1	0,00	0	5,0	24,61	100	0	0	92	3	2	1	1	0	0	0	0	0	0	0	1
75	0	0,00	0	5,1	26,04		0	0	96	6	2	1	1	0	0	0	0	0	0	0	0
65	1	0,00	0	5,4	27,39	89	0	0	121	0	2	1	1	0	0	0	1	1	0	0	0
86	0	0,00	0	5,3	27,06	125	0	0	86	11	2	1	1	0	0	0	0	0	0	0	0
83	0	0,00	0	5,4	32,00	110	0	0	103	5	2	1	0		0	0	1	0	0	0	2
55	1	0,00	0	5,5	27,10	87	0	0	124	5	2	0	1	0	0	0	0	1	0	0	0
51	1	0,00	0	4,6	23,44	104	0	0	182	4	2	0	0	0	1	0	1	1	0	0	0
58	0	1,00	1	5,9	25,39	100	0	0	151	4	2	1	0	0	0	0	0	0	0	0	0
71	1	1,00	1	5,4	29,38	107	0	0	78	3	2	1	0	1	0	0	0	0	0	0	0
73	1	1,00	1	5,4	22,48	87	0	0	108	5	2	1	0	0	0	0	0	0	0	0	0
78	0	1,00	1	5,4	31,64	119	0	0	95	6	2	1	0	0	0	0	0	0	0	0	0
81	1	1,00	1	5,7	29,34	113	0	0	113	1	2	0	1	1	0	0	0	0	0	0	2
82	0	1,00	1	5,4	26,95	100	0	0	104	3	2	1	0	0	0	0	0	0	0	0	0
80	0	1,00	1	5,8	26,38	83	0	0	97	3	2	1	0	0	0	0	0	0	0	0	0
46	1	1,00	1	5,9	27,04	100	0	0	232	0	2	0	0	0	0	0	0	0	0	0	0
60	1	1,00	1	5,7	31,14	115	0	0	81	1	2	0	1	1	1	0	1	1	0	0	0
64	1	1,00	1	5,6	22,60	80	0	0	101	2	2	0	0	0	0	0	0	0	0	0	0
67	1	1,00	1	6,0			0	0	131	1	2	1	0	0	0	0	1		0	0	0
51	0	1,00	1	5,3	31,11	98	0	0	110	4	2	1	0	0	0	0	0	1	0	0	0
65	0	1,00	1	5,8	27,70	102	0	0	129	1	2	1	0	0	0	0	0	0	0	0	0
59	1	1,00	1	5,5		101	0	0	130	2	2	0	0	0	0	0	1	1	0	0	0
82	0	1,00	1	5,9			0	0	99	4	2	1	0	0	0	0	0	0	0	0	0
46	1	1,00	1	6,3	23,67		0	0	119	3	2	1	0	0	0	0	0	1	0	0	0
65	1	1,00	1	6,3	29,24	100	0	0	103	0	2	0	1	0	0	0	0	1	0	0	0
65	1	1,00	1	5,8			0	0	147	10	2	1	1	0	0	0	0	1	0	0	0
49	1	1,00	1	5,8	25,86		0	0	126	2	2	0	0	0	0	0	0	1	0	0	0
73	1	1,00	1	5,9	31,10	107	0	0	113	3	2	1	1	0	0	0	1	0	0	0	0
49	1	1,00	1	6,2	28,60		0	0	86	1	2	1	1	0	0	0	1	1	0	0	0
84	1	1,00	1	6,2	24,57	93	0	0	132	2	2	1	0	1	0	0	0	0	0	0	3
68	1	1,00	1	5,8	26,73	103	0	0	92	1	2	0	1	0	0	0	0	0	0	0	0
80	0	1,00	1	6,2	24,20	87	0	0	122	2	2	1	1	0	0	0	0	0	0	0	0

81	1	1,00	1	5,8	21,45		0	0	101	3	2	1	0	0	0	0	0	0	0	0	0
88	0	1,00	1	5,7			0	0	124	3	2	1	1	0	0	0	0	0	0	0	0
90	0	1,00	1	6,0	24,98	83	0	0	122	3	2	1	0	0	0	0	0	0	0	0	2
78	0	1,00	1	6,1	29,38	112	0	0	110	3	2	1	1	0	0	0	1	0	0	0	0
79	0	1,00	1	5,9	22,27	97	0	0	125	3	2	1	1	0	0	0	0	0	0	0	1
77	1	1,00	1	5,9	30,85	108	0	0	99	3	2	0	0	0	0	0	1	0	0	0	0
66	1	1,00	1	5,7	24,51		0	0	94	3	2	1	0	1		0	1	1	0	0	0
86	1	1,00	1	6,3	27,64	102	0	0	180	1	2	1	0	0	1	0	0	0	0	0	2
79	0	1,00	1	5,7	26,02	92	0	0	127	3	2	1	1	0	0	0	0	0	0	0	3
76	0	1,00	1	5,7	27,34	100	0	0	92	1	2	1	1	0	0	0	0	0	0	0	0
60	1	1,00	1	5,9	30,47	101	0	0	101	5	2	0	0	0	0	0	0	1	0	0	2
44	0	1,00	1	5,8	23,11	80	0	0	85	1	2	1	0	0	0	0	0	0	0	0	0
83	0	1,00	1	6,3	29,52	103	0	0	84	6	2	1	0	0	0	0	0	0	0	0	0
68	1	1,00	1	5,9	37,38	122	0	0	107	2	2	1	0	0	0	0	0	0	0	0	0
77	0	1,00	1	5,9	27,47	93	0	0	96	9	2	1	1	1	0	0	0	0	0	0	0
59	0	1,00	1	5,8	41,09	128	0	0	135	4	2	1	0	0	0	0	1	0	0	0	2
78	1	1,00	1	6,0	21,97	101	0	0	118	5	2	0	1	0	0	0	1	1	0	0	0
86	1	1,00	1	5,9	17,36		0	0	112	3	2	1	1	0	0	0	1	0	0	0	0
69	1	1,00	1	5,8	31,27		0	0	112	3	2	1	1	0	0	0	0	0	0	0	1
78	0	1,00	1	6,4	32,85	116	0	0	161	1	2	1	1	0	1	0	0	0	0	0	0
77	1	1,00	1	5,7	33,06	106	0	0	115	4	2	1	0	0	0	0	0	0	0	0	0
83	0	1,00	1	5,7	26,04	92	0	0	101	5	2	1	1	0	0	0	0	0	0	0	0
71	1	1,00	1	6,2	25,26	105	0	0	100	4	2	1	1	0	0	0	0	0	0	0	0
59	1	1,00	1	6,2	26,17	94	0	0	106	3	2	0	1	0	0	0	1	1	0	0	0
55	1	1,00	1	5,7			0	0	135	4	2	1	1	0	0	0	1	0	0	0	0
71	0	1,00	1	5,8	25,00	94	0	0	99	2	2	1	1	0	0	0	0	0	0	0	0
68	0	1,00	1	6,1	37,89	122	0	0	96	1	2	1	1	0	0	0	0	0	0	0	1
81	1	1,00	1	5,9	25,33		0	0	108	4	2	1	1	0	0	0	0	0	0	0	0
78	0	1,00	1	5,8	31,64	155	0	0	106	0	2	1	0	0	0	0	0	0	0	0	0
52	0	1,00	1	6,1	24,22	90	0	0	136	3	2	1	1	0	0	0	0	0	0	0	0
66	0	1,00	1	5,7	19,88	100	0	0	103	3	2	1	1	0	1	0	0	1	0	0	0
54	0	1,00	1	5,9	31,63		0	0	91	0	2	1	0	0	0	0	0	0	0	0	0
55	1	1,00	1	5,9	31,02	110	0	0	115	4	2	1	0	0	0	0	1	1	0	0	0
78	1	1,00	1	5,7	26,06		0	0	111	2	2	1	1	0	0	0	0	0	0	0	0
66	1	1,00	1	5,8	20,96	55	0	0	103	1	2	0	0	0	0	0	0	1	0	0	0
77	1	1,00	1	6,2	28,73		0	0	185	1	2	0	1		0	0	0	1	0	0	0
62	0	1,00	1	5,8	33,20	130	0	0	120	4	2	0	1	0	0	0	0	0	0	0	0

81	0	1,00	1	5,9	36,98	116	0	0	101	2	2	0	0	0	0	0	0	0	0	0	0
71	1	2,00	2	14,8			1	1	264	3	2	1	1	0	0	0		0	0	0	0
69	1	2,00	2	7,8	28,33	108	1	1	182	18	2	1	0	0	0	0	0	0	0	0	1
65	0	2,00	2	6,5	29,76	103	1	1	163	1	2	1	1	0	0	0	0	0	0	0	0
80	1	2,00	2	6,7	29,39	103	1	1	132	14	2	1	0	0	0	0			0	0	2
57	1	2,00	2	9,6	25,86	94	1	1	239	5	2	0	0	0	0	0	0	1	0	0	0
78	0	2,00	2	7,1	29,13	110	1	1	183	8	2	1	0	0	0	0	0	0	0	0	0
57	1	2,00	2	7,2	24,51	82	1	1	106	1	2	0	0	0	0	0	1	1	0	0	0
92	1	2,00	2	8,1			1	1	141	7	2	1	1	0	1	0	0	0	0	0	1
82	0	2,00	2	6,5	17,04	108	1	1	110	3	2	0	1	0	0	0	0	0	0	0	3
78	0	2,00	2	6,8			1	1	98	5	2	1	0	0	0	0	0	0	0	0	1
61	0	2,00	2	14,5	24,44	74	1	1	402	3	2	0	0	0	0	0	0	0	0	0	0
76	0	2,00	2	6,8	30,82	141	1	1	111	6	2	1	1	0	0	0	0	0	0	0	3
67	1	2,00	2	7,2	33,46	105	1	1	147	3	2	1	0	0	0	0	0	0	0	0	2
63	0	2,00	2	7,0	30,00	112	1	1	163	3	2	0	0	0	0	0	1	1	0	0	0
80	1	2,00	2	6,7	25,61		1	1	143	2	2	0	1	0	1	0	0	0	0	0	0
49	1	2,00	2	7,2	26,04	112	1	1	146	5	2	1	1	0	0	0	1	1	0	0	0
74	0	2,00	2	7,0	27,06	98	1	1	145	5	2	1	1	0	0	0	0	0	0	0	0
73	1	0,00	2	6,7			1	1	140	5	2	1	0	0	0	0	1	0	0	0	1
82	0	0,00	2	6,6	35,11	106	1	1	90	4	2	1	1	0	0	0	0	0	0	0	0
69	0	0,00	0	5,0	32,89	105	0	0	114	5	3	1		0	0	1	1	0	0	0	0
82	0	0,00	0	5,2	23,23	90	0	0	149	5	3	1	0	0	1	1	0	0	0	0	1
90	0	0,00	0	5,1	23,59	90	0	0	116	20	3	1	0	0	0	1	0	0	0	0	0
80	0	0,00	0	4,8	23,78	78	0	0	145	16	3	0	0	0	0	0	0	0	0	0	0
82	0	0,00	0	5,0	23,44	107	0	0	95	5	3	0	0	0	0	1	0	0	0	0	0
78	0	0,00	0	5,0	37,78	126	0	0	136	15	3	1	1	0	0	1	0	0	0	0	3
85	1	0,00	0	4,3	24,22	81	0	0	101	10	3	0	0	0	1	1	0	0	0	0	0
77	0	0,00	0	4,5	22,58	85	0	0	108	7	3	1	0	0	0	1	0	0	0	0	0
46	0	0,00	0	4,8	29,14	104	0	0	135	13	3	0	0	0	0	0	1	1	0	0	0
76	0	0,00	0	4,8	24,77	89	0	0	120	21	3	1	0	0	0	1	0	0	0	0	0
71	1	0,00	0	4,4	26,83	102	0	0	95	2	3	1	1	0	1	0	0	0	0	0	0
83	0	0,00	0	5,1	21,48	98	0	0	103	3	3	1	0	0	0	1	0	0	0	0	0
69	0	0,00	0	4,8	25,61	96	0	0	127	8	3	0	1	0	0	1	0	0	0	0	0
79	0	0,00	0	4,7	27,48	107	0	0	100	5	3	1	1	0	1	1	0	0	0	0	0
77	0	0,00	0	4,6	21,79	102	0	0	117	13	3	1	0	0	0	1	0	0	0	0	0
74	0	0,00	0	5,0	25,81	101	0	0	123	4	3	1	1	0	1	1	0	0	0	0	0
86	0	0,00	0	5,1	24,97	83	0	0	95	6	3	1	0	0	0	1	0	0	0	0	0

93	0	0,00	0	5,1	30,48	80	0	0	114	8	3	0	0	0	0	0	0	0	0	0	0
80	1	0,00	0	5,6	23,45	101	0	0	101	3	3	1	1	1	0	1	0	0	0	0	0
82	0	0,00	0	5,1	18,51	82	0	0	111	6	3	1	0	0	1	1	0	0	0	0	0
88	1	0,00	0	5,2	21,85	100	0	0	127	7	3	0	1	0	0	1	1	0	0	0	0
68	0	0,00	0	4,6	23,88	89	0	0	100	2	3	1	1	0	0	0	0	0	0	0	1
84	0	0,00	0	4,9		82	0	0	125	4	3	0	0	0	0	1	0	0	0	0	2
80	0	0,00	0	4,6	26,16	86	0	0	119	9	3	0	0	0	0	1	0	0	0	0	0
70	0	0,00	0	5,5	29,30	100	0	0	91	3	3	1	1	0	0	1	0	0	0	0	1
82	0	0,00	0	4,9	22,77	97	0	0	76	5	3	1	0	0	0	1	0	0	0	0	0
77	1	0,00	0	5,2	22,58	95	0	0	111	25	3	0	0	1	0	1	1	1	0	0	3
86	1	0,00	0	5,2	27,68	105	0	0	106	4	3	1	1	0	0	1	0	0	0	0	0
84	0	0,00	0	4,8	19,65	92	0	0	114	3	3	1	1	0	0	0	0	0	0	0	0
65	0	0,00	0	4,8	38,10	126	0	0	114	5	3	1	1	0	0	1	0	0	0	0	0
79	1	0,00	0	5,0	24,57	98	0	0	186	9	3	1	0	0	0	1	0	0	0	0	0
88	1	0,00	0	5,4	18,37	80	0	0	139	1	3	1	0	0	1	1	0	0	0	0	0
79	0	0,00	0	5,1	33,33	100	0	0	142	5	3	1	0	0	0	1	0	0	0	0	0
76	0	0,00	0	4,3	32,87	101	0	0	101	5	3	1	0	0	0	1	0	0	0	0	3
79	0	0,00	0	5,2			0	0	114	12	3	0	1	1	0	1	0	0	0	0	0
82	0	0,00	0	4,7	24,44	103	0	0	140	3	3	1	0	0	0	1	0	0	0	0	2
72	1	0,00	0	5,1	23,61	86	0	0	104	3	3	1	0	0		1	1	0	0	0	0
82	0	0,00	0	4,7	23,14	78	0	0	89	11	3	0	0	0	0	0	0	0	0	0	1
83	0	0,00	0	4,7	27,53	76	0	0	107	12	3	0	0	0	0	1	0	0	0	0	1
75	1	0,00	0	5,3	26,26	108	0	0	113	12	3	0	0	0	0	1	0	0	0	0	0
63	0	0,00	0	5,1	30,67	83	0	0	115	4	3	1	0	0	0	1	0	0	0	0	1
92	1	0,00	0	4,9	28,29	112	0	0	115	5	3	1	0	0	1	0	0	0	0	0	3
83	0	0,00	0	4,7	23,92	83	0	0	85	7	3	1	1	0	0	1	0	0	0	0	3
83	0	0,00	0	4,9	36,52		0	0	133	20	3	1	0	0	0	1	0	0	0	0	0
73	1	0,00	0	4,7	27,43	100	0	0	99	7	3	0	0	0	0	0	0	0	0	0	0
88	1	0,00	0	5,0	22,15	90	0	0	97	4	3	0	0	0	0	1	0	0	0	0	0
83	0	0,00	0	5,5	32,89	97	0	0	140	21	3	1	1	0	0	1	0	0	0	0	0
78	0	0,00	0	4,7	28,13	88	0	0	100	8	3	0	0	0	0	0	0	0	0	0	0
59	1	0,00	0	4,9	27,55	83	0	0	107	5	3	0	0	0	0	1	0	1	0	0	0
85	1	0,00	0	5,3	31,25	100	0	0	198	11	3	0	0	0	0	1	0	0	0	0	0
77	0	0,00	0	4,7	36,05	106	0	0	106	5	3	1	0	0	1	1	0	0	0	0	0
85	1	0,00	0	4,5	20,54	73	0	0	89	6	3	0	1	0	0	1	1	1	0	0	0
90	0	0,00	0	5,2	33,23	96	0	0	117	20	3	1	0	0	0	1	0	0	0	0	0
82	0	0,00	0	4,9	30,47	104	0	0	98	6	3	1	1	1	0	1	0	0	0	0	0

79	1	0,00	0	4,0	29,23	116	0	0	95	9	3	1	0	0	0	1	1	1	0	0	2
80	1	0,00	0	4,8	30,90	90	0	0	115	13	3	1	0	0	0	0	0	0	0	0	0
90	1	0,00	0	4,8	24,03	80	0	0	112	8	3	0	0	0	0	0	1	1	0	0	0
85	0	0,00	0	5,2	28,89	94	0	0	156	18	3	1	0	0	0	1	0	0	0	0	0
55	0	0,00	0	5,2	25,97	82	0	0	98	5	3	0	0	0	0	1	0	0	0	0	1
73	0	0,00	0	4,8	27,03	103	0	0	90	16	3	0	0	0	0	1	0	0	0	0	0
86	0	0,00	0	4,7			0	0	131	19	3	1	0	1	0	1	0	0	0	0	0
88	0	0,00	0	4,6	22,22	65	0	0	104	16	3	1		0	0	1	0	0	0	0	3
87	0	0,00	0	4,9	25,71	85	0	0	95	3	3	1	0	0	0	1	0	0	0	0	0
84	1	0,00	0	5,2			0	0	130	4	3	1	0	0	0	0	0	1	0	0	1
42	0	0,00	0	4,4	24,34	90	0	0	92	13	3	1	0	0	1	0	1	1	0	0	0
68	0	0,00	0	5,2			0	0	148	8	3	1	0	0	0	1	0	0	0	0	0
76	0	0,00	0	5,2	24,82	83	0	0	138	21	3	0	0	0	0	1	0	0	0	0	0
85	1	0,00	0	5,6			0	0	98	7	3	1	0	0	1	1	0	0	0	0	2
82	0	0,00	0	5,5			0	0	99	7	3	1	1	0	0	1	0		0	0	3
73	0	0,00	0	4,8	23,74	109	0	0	106	1	3	1	0	0	0	1	0	0	0	0	0
55	1	0,00	0	5,5	39,89	130	0	0	96	0	3	1	0	0	0	0	0	1	0	0	0
85	0	0,00	0	5,2	20,00		0	0	100	2	3	1	0	0	0	1	0	0	0	0	0
81	0	0,00	0	5,0	30,04	98	0	0	114	3	3	0	1	0	0	1	0	0	0	0	3
98	0	0,00	0	4,7			0	0	125	20	3	1	0	0	0	1	0	0	0	0	3
64	0	0,00	0	5,0	26,14		0	0	120	20	3	0	0	0	0	1	0	0	0	0	0
82	0	0,00	0	5,0			0	0	90	10	3	1	0	0	0	1	0	0	0	0	3
73	1	0,00	0	5,3	25,46		0	0	81	6	3	1	1	1	0	1	0	1	0	0	0
86	0	0,00	0	5,5			0	0	137	3	3	1	0	0	0	0	0	0	0	0	0
54	1	0,00	0	5,2	28,41		0	0	118	12	3	1	0	0	0	0	1	1	0	0	0
85	0	0,00	0	5,5	27,34	88	0	0	145	5	3	1	0	0	0	1	1	0	0	0	3
87	0	0,00	0	5,6			0	0	122	20	3	1	0	0	0	1	0	0	0	0	0
74	1	0,00	0	5,5	25,51	108	0	0	182	6	3	0	1	0	0	1	0	0	0	0	0
93	0	0,00	0	5,6	24,00	79	0	0	138	18	3	0	0	0	0	1	0	0	0	0	0
76	0	0,00	0	5,5			0	0	103	3	3	1	1	0	0	1			0	0	0
74	0	0,00	0	5,5	19,81	72	0	0	84	9	3	1	0	0	1	0		0	0	0	0
82	0	0,00	0	5,3	36,44	115	0	0	87	7	3	1	0	0	0	1	0	0	0	0	1
70	0	0,00	0	5,2	29,27	110	0	0	83	18	3	1	0	0	0	1	0	0	0	0	0
73	0	0,00	0	5,5	34,48	130	0	0	144	15	3	1	1	0	0	1	0	0	0	0	0
75	1	0,00	0	5,6	29,38	110	0	0	194	3	3	1	0	0	0	1	1	0	0	0	2
29	1	0,00	0	5,2	24,34	89	0	0	104	3	3	1	0	1	0	0	0	0	0	0	0
73	0	0,00	0	5,6	25,39	95	0	0	173	4	3	1	1	0	0	1	0	0	0	0	0

60	1	0,00	0	5,3	24,22	83	0	0	91	15	3	0	1	0	0	1	0	0	0	0	0
65	1	0,00	0	5,5	29,75	105	0	0	91	3	3	0	1	0	0	1	0	0	0	0	0
79	0	0,00	0	5,6	22,77	98	0	0	114	19	3	1	1	0	0	1	0	0	0	0	0
82	0	0,00	0	5,6	27,11	100	0	0	104	4	3	1	1	0	1	1	0	0	0	0	0
83	1	0,00	0	5,4	22,50	93	0	0	114	8	3	1	0	0	0	0	0	0	0	0	0
63	1	0,00	0	5,4		103	0	0	116	11	3	0	0	0	0	1	0	1	0	0	1
66	1	0,00	0	5,0	27,76	99	0	0	115	14	3	1	1	0	0	1	0	1	0	0	0
76	1	0,00	0	5,4	28,23	408	0	0	93	21	3	0	0	0	0	1	0	0	0	0	0
81	0	0,00	0	5,6	26,49	97	0	0	115	3	3	1	1	0	0	1	0	0	0	0	3
64	0	0,00	0	4,9	23,74	63	0	0	107	9	3	0	0	0	0	1	0	0	0	0	3
88	0	0,00	0	4,9	24,39	83	0	0	94	6	3	1	0	0	0	1	0	0	0	0	2
83	1	0,00	0	5,6	27,68	101	0	0	106	21	3	1	0	0	1	1	0	0	0	0	0
86	0	0,00	0	5,4	25,78	89	0	0	85	19	3	0	1	0	0	1	0	0	0	0	2
83	0	0,00	0	5,1	22,64		0	0	105	3	3	1	0	0	0	1	0	0	0	0	0
79	0	0,00	0	5,4	26,40	94	0	0	97	2	3	1	1	0	0	1	0	0	0	0	1
63	1	0,00	0	5,0	26,23	110	0	0	145	16	3	0	1	0	0	1	0	0	0	0	0
82	1	0,00	0	5,6	24,46	95	0	0	154	10	3	1	1	0	1	0	0	0	0	0	1
81	0	0,00	0	5,2	26,67	89	0	0	92	6	3	0	1	0	0	1	0	0	0	0	1
56	1	0,00	0	5,3	39,25	150	0	0	108	20	3	0	0	0	0	0	1	0	0	0	0
74	0	0,00	0	5,6		100	0	0	123	23	3	1	0	0	0	1	0	0	0	0	0
78	1	0,00	0	5,6	27,78		0	0	127	6	3	1	0	0	0	1	1	0	0	0	0
65	1	0,00	0	5,3	23,59	90	0	0	103	23	3	1	0	0	0	1	1	0	0	0	0
80	0	0,00	0	5,1	27,48	105	0	0	167	13	3	1	0	1	0	1	0	0	0	0	3
47	1	0,00	0	5,2	24,26		0	0	140	6	3	1	0	0	0	1	1	1	0	0	0
85	1	1,00	1	6,3	25,06	101	0	0	199	8	3	1	0	0	0	1	0	0	0	0	3
68	1	1,00	1	5,5	34,02	125	0	0	113	3	3	1	0	0	0	1	0	0	0	0	0
89	0	1,00	1	5,7	25,89	93	0	0	163	6	3	1	1	0	0	1	0	0	0	0	1
81	0	1,00	1	5,5	18,73	80	0	0	98	19	3	1	1	0	0	1	0	0	0	0	1
78	0	1,00	1	5,4	25,78	108	0	0	131	5	3	1	0	0	0	1	0	0	0	0	0
71	1	1,00	1	5,4	20,20	88	0	0	128	3	3	1	1	0	1	0	0	1	0	0	0
59	1	1,00	1	5,6	20,98	84	0	0	114	19	3	1	1	0	1	0	0	0	0	0	0
91	0	1,00	1	5,6	35,09	112	0	0	132	11	3	1	0	0	1	1	0	0	0	0	0
78	1	1,00	1	5,3	25,95	102	0	0	115	4	3	1	0	0	0	1	0	0	0	0	0
69	0	1,00	1	5,3	30,12	116	0	0	91	8	3	0	0	0	0	1	0	0	0	0	0
83	1	1,00	1	5,4	29,97	92	0	0	130	18	3	1	0	0	0	1	1	1	0	0	0
88	1	1,00	1	6,1	21,45		0	0	133	5	3	1	1	0	0	1	0	0	0	0	1
69	1	1,00	1	5,9	26,30	97	0	0	148	5	3	0	0	0	0	0	0	1	0	0	0

69	0	1,00	1	5,4	27,06	92	0	0	124	4	3	1	0	0	0	1	0	0	0	0	0
83	1	1,00	1	5,8	32,05	98	0	0	132	2	3	1	0	0	0	1	1	0	0	0	0
50	1	1,00	1	5,5	28,68		0	0	147	1	3	0	1	0	1	0	0	1	0	0	1
74	0	1,00	1	5,7	30,83	116	0	0	112	1	3	1	0	0	0	1	0	0	0	0	0
75	0	1,00	1	5,6	40,00	104	0	0	121	3	3	1	1	0	0	1	0	0	0	0	0
79	0	1,00	1	5,5	27,68	110	0	0	111	13	3	0	0	0	0	0	0	0	0	0	2
66	0	1,00	1	5,8	25,34	98	0	0	124	18	3	1	1	0	0	1	1	0	0	0	0
77	0	1,00	1	6,2	26,49	98	0	0	214	18	3	1	0	0	0	1	0	0	0	0	3
79	0	1,00	1	5,8	28,58		0	0	130	21	3	1	1	0	0	1	0	0	0	0	0
70	1	1,00	1	5,9	26,72	99	0	0	123	7	3	1	1	0	0	1	1	0	0	0	0
76	0	1,00	1	5,3	29,55		0	0	98	12	3	1	0	0	0	1	0	0	0	0	3
80	0	1,00	1	6,0	33,20	113	0	0	107	11	3	1	1	0	1	1	0	0	0	0	0
92	1	1,00	1	5,9	22,37	80	0	0	89	8	3	0	0	0	0	1	1	0	0	0	0
88	0	1,00	1	6,0	29,90	94	0	0	128	13	3	0	0	0	0	1	0	0	0	0	0
63	0	1,00	1	5,8	32,89	106	0	0	107	14	3	0	1	0	0	1	0	0	0	0	0
66	0	1,00	1	5,8	35,06	110	0	0	165	7	3	0	0	0	0	1	0	0	0	0	0
78	0	1,00	1	6,0	24,97	80	0	0	102	17	3	1	1	0	0	1	0	0	0	0	1
77	0	1,00	1	5,8	27,34	110	0	0	135	15	3	1	1	0	1	1	0	0	0	0	0
75	0	1,00	1	5,8	27,82		0	0	101	12	3	1	0	0	0	1	0	0	0	0	0
87	0	1,00	1	5,9	42,67	80	0	0	115	4	3	1	1	0	0	1	0	0	0	0	3
67	0	1,00	1	5,8	30,08	97	0	0	107	3	3	1	1	0	1	1	0	0	0	0	1
82	0	1,00	1	6,0	28,69	93	0	0	138	17	3	0	0	0	0	1	0	0	0	0	0
49	1	1,00	1	6,0	24,06		0	0	107	2	3	0	0	0	0	0	1	1	0	0	0
65	1	1,00	1	5,9	29,39	112	0	0	122	23	3	0	0	0	0	1	0	0	0	0	0
90	0	1,00	1	5,7			0	0	112	2	3	1	1	1	0	1	0	0	0	0	1
85	0	1,00	1	5,8	23,37		0	0	149	3	3	1	0	0	0	1	0		0	0	1
79	0	1,00	1	5,8	25,43	100	0	0	84	5	3	1	1	0	0	1	0	0	0	0	1
80	0	1,00	1	6,0	38,22	126	0	0	123	5	3	1	0	0	0	1	0	0	0	0	1
84	0	1,00	1	5,8	24,44	92	0	0	190	3	3	1	1	1	0	1	0	0	0	0	3
82	0	1,00	1	6,0	31,25	98	0	0	120	19	3	1	1	0	0	1	0	0	0	0	1
82	1	1,00	1	5,8			0	0	108	22	3	1	1	0	0	1	0	0	0	0	3
78	0	1,00	1	6,0	26,73	96	0	0	108	20	3	0	0	0	0	1	0	0	0	0	3
61	1	1,00	1	5,9	28,91	97	0	0	108	19	3	1	1	0	0	1	0	0	0	0	0
56	0	1,00	1	6,3	36,72	113	0	0	127	17	3	1	0	0	0	1	1	1	0	0	0
86	0	1,00	1	6,0	30,18	104	0	0	128	3	3	0	0	0	0	1	0	0	0	0	0
87	0	1,00	1	6,3	24,44	97	0	0	157	8	3	1	0	0	1	1	0	0	0	0	1
87	0	1,00	1	5,8	21,78	90	0	0	126	5	3	1	0	0	0	1	0	0	0	0	0

65	1	1,00	1	5,9	31,83	115	0	0	124	13	3	1	0	0	0	1	0	1	0	0	0
64	0	1,00	1	5,7	24,01		0	0	164	19	3	1	1	0	0	1	0	0	0	0	0
82	1	1,00	1	5,8	29,68	120	0	0	129	1	3	1	0	1	0	1	1	0	0	0	3
84	1	1,00	1	5,9	25,95	96	0	0	119	10	3	1	1	0	0	1	0	0	0	0	0
87	0	1,00	1	6,4	33,56	109	0	0	133	0	3	1	1	0	0	1	0	0	0	0	1
68	0	1,00	1	6,4	26,06		0	0	93	3	3	1	1	0	0	0	0	0	0	0	0
74	0	1,00	1	6,0	21,87	80	0	0	126	21	3	0	1	0	0	1	0	1	0	0	0
83	1	1,00	1	6,0	28,52	100	0	0	128	20	3	0	1	0	0	1	0	0	0	0	1
51	0	1,00	1	5,8	21,30	87	0	0	138	19	3	1	0	0	0	1	1	0	0	0	0
84	0	1,00	1	6,3		155	0	0	156	13	3	1	0	0	0	1	0	0	0	0	0
83	0	1,00	1	5,9		75	0	0	137	6	3	1	0	0	0	1	0	0	0	0	0
90	0	1,00	1	6,1	24,26	101	0	0	93	8	3	1	0	0	0	1	0	0	0	0	3
76	0	1,00	1	5,7	23,44	120	0	0	93	1	3	1	1	0	0	1	0	0	0	0	0
81	1	1,00	1	5,7	24,80	97	0	0	121	4	3	1	0	0	0	1	0	0	0	0	0
82	0	1,00	1	5,7	20,00		0	0	111	21	3	1	1	0	0	1	0	0	0	0	0
74	0	1,00	1	6,1	37,58	150	0	0	108	8	3	1	1	0	0	1	0	0	0	0	0
76	0	1,00	1	5,9	25,14	102	0	0	120	0	3	1	0	0	0	1	0	0	0	0	2
55	1	1,00	1	6,2	22,06	86	0	0	105	17	3	0	0	0	1	1	0	1	0	0	1
84	0	1,00	1	5,8			0	0	96	12	3	0	0	0	0	1	0	0	0	0	2
84	0	1,00	1	5,7	33,33	112	0	0	93	2	3	1	1	0	0	1	0	0	0	0	3
68	1	1,00	1	6,3	27,04		0	0	148	1	3	1	0	0	0	1	0	0	0	0	0
77	0	1,00	1	5,9	20,20	70	0	0	91	2	3	0	0	0	1	1	0	0	0	0	2
83	1	1,00	1	5,9	27,55		0	0	124	6	3	1	0	0	0	1	0	0	0	0	0
90	0	1,00	1	5,7	25,39	80	0	0	113	2	3	1	0	0	0	1	0	0	0	0	2
94	1	1,00	1	5,7	23,11		0	0	91	7	3	1	0	0	0	1	1	1	0	0	2
92	0	1,00	1	6,0	24,22	110	0	0	235	10	3	1	0	0	0	1	0	0	0	0	0
87	0	1,00	1	5,9	26,67	105	0	0	110	2	3	1	1	0	0	1	0	0	0	0	0
84	0	5,00	2	6,5	31,25	98	1	1	128	9	3	1	0	0	0	1	0	0	0	0	3
54	0	4,00	2	7,0	37,02	91	1	1	151	2	3	0	1	0	1	1	1	0	0	0	1
62	0	3,00	2	6,6	30,30	100	1	1	138	19	3	0	0	0	1	1	0	0	0	0	3
58	1	2,00	2	8,1	26,75	116	1	1	224	3	3	0	0	0	0	0	1	1	0	0	0
59	1	2,00	2	7,8			1	1	128	2	3	1	1	0	1	0	0	0	0	0	0
76	0	2,00	2	7,3			1	1	159	4	3	1	0	1	0	1	0	0	0	0	0
77	0	2,00	2	7,9	24,97	88	1	1	201	22	3	1	1	0	0	0	0	0	0	0	0
82	0	2,00	2	8,1			1	1	132	8	3	1	0	0	0	1	0	0	0	0	3
83	1	2,00	2	6,8	28,37	118	1	1	161	4	3	1	0	0	0	0	0	0	0	0	2
77	0	2,00	2	7,3	31,64	98	1	1	96	9	3	1	1	0	0	0	0	0	0	0	2

76	1	2,00	2	7,9		129	1	1	235	15	3	1	1	0	0	1	0	0	0	0	1
81	0	2,00	2	6,8	39,13	116	1	1	110	4	3	1	1	0	1	1	0	0	0	0	3
73	0	2,00	2	6,5			1	1	155	19	3	1	0	0	0	1	0	0	0	0	0
83	1	2,00	2	6,6	23,94	92	1	1	148	6	3	1	0	0	0	1	0	1	0	0	0
93	0	2,00	2	6,7	22,66	77	1	1	113	14	3	1	1	0	0	1	0	0	0	0	1
69	0	2,00	2	7,2	29,30	109	1	1	125	8	3	0	0	0	1	1	0	0	0	0	1
76	1	2,00	2	7,1	31,25	98	1	1	122	18	3	0	1	1	1	1	0	0	0	0	1
75	0	2,00	2	6,6	34,11	92	1	1	85	4	3	1	0	0	0	1	0	0	0	0	3
53	0	2,00	2	7,0	34,93	110	1	1	188	16	3	0	0	0	0	1	0	0	0	0	0
74	1	2,00	2	6,7	27,18		1	1	197	3	3	1	0	1	1	1	0	0	0	0	1
87	0	2,00	2	9,6			1	1	328	1	3	0	1	0	0	1	0	0	0	0	0
77	0	0,00	2	6,6	30,36	120	1	1	123	17	3	1	0	0	0	1	0	0	0	0	1
75	0	0,00	2	7,0	28,34	108	1	1	196	4	3	0	1	0	0	1	0	0	0	0	0
84	1	0,00	2	6,5		113	1	1	117	10	3	0	1	0	0	1	0	0	0	0	0
62	0	0,00	2	6,5	34,17	112	1	1	148	7	3	0	0	0	0	1	0	0	0	0	0
66	0	0,00	2	6,5	24,35		1	1	171	17	3	1	1	0	0	1	0	0	0	0	0
65	0	0,00	2	6,7	23,12	86	1	1	130	3	3	1	1	0	0	1	0	0	0	0	0
84	1	0,00	2	6,9	29,14	90	1	1	128	2	3	1	1		0	1	0	0	0	0	0
85	0	0,00	2	6,5	29,30	100	1	1	140	21	3	1	0	0	0	1	0	0	0	0	1
85	0	0,00	2	6,5	29,00	102	1	1	113	2	3	1	0	0	0	1	0	0	0	0	1
82	1	0,00	0	5,2	21,63	86	0	0	102	4	4	0	0	0	0	0	0	1	0	0	0
58	1	0,00	0	4,5	22,84	90	0	0	115	0	4	0	1	0	0	0	1	1	0	0	0
60	1	0,00	0	4,9	21,80	88	0	0	123	18	4	1	1	0	1	0	0	0	0	0	0
81	1	0,00	0	4,7	26,81	100	0	0	105	7	4	1	1	0	1	0	0	0	0	0	0
71	1	0,00	0	5,0	31,22	100	0	0	67	2	4	0	0	0	0	1	0	1	0	0	0
72	0	0,00	0	5,2	25,71	86	0	0	119	2	4	0	0	0	0	0	0	1	0	0	0
76	0	0,00	0	4,5	26,35	90	0	0	89	6	4	1	0	0	0	0	0	0	0	0	0
83	1	0,00	0	4,9	20,20	84	0	0	135	8	4	1	1	0	1	1	0	0	0	0	0
77	0	0,00	0	5,1	33,87	106	0	0	122	11	4	0	0	0	0	0	0	0	0	0	0
86	1	0,00	0	5,0	24,80	98	0	0	116	4	4	0	0	0	0	0	0	0	0	0	0
76	1	0,00	0	4,4	28,73	110	0	0	108	8	4	0	0	0	0	0	0	0	0	0	1
88	0	0,00	0	4,9	22,04	92	0	0	94	4	4	1	0	0	0	0	0	0	0	0	0
68	0	0,00	0	4,7	23,78	100	0	0	98	6	4	1	1	0	0	0	0	0	0	0	0
68	1	0,00	0	4,9	26,45	92	0	0	101	1	4	1	1	0	0	0	1	0	0	0	0
88	0	0,00	0	4,7	27,56	99	0	0	92	6	4	1	1	0	0	0	0	0	0	0	3
70	0	0,00	0	4,9	24,09	102	0	0	118	4	4	1	0	0	0	0	0	0	0	0	0
89	0	0,00	0	5,0	25,11		0	0	147	14	4	1	0	0	0	0	0	0	0	0	0

42	1	0,00	0	4,3			0	0	83	1	4	0	1	0	0	0	0	1	0	0	0
46	1	0,00	0	5,2	17,58	76	0	0	178	10	4	1	0	0	0	0	0	0	0	0	0
79	0	0,00	0	4,8	33,33	103	0	0	128	0	4	1	1	0	0	0	0	0	0	0	1
85	0	0,00	0	4,6	20,40	63	0	0	85	2	4	1	0	0	0	0	0	0	0	0	1
97	0	0,00	0	4,3	22,89	95	0	0	116	12	4	1	0	0	1	0	0	0	0	0	3
86	1	0,00	0	4,7	23,53	82	0	0	96	2	4	0	0	0	0	0	0	0	0	0	0
52	1	0,00	0	4,7	26,87	111	0	0	106	5	4	0	0	0	0	0	0	1	0	0	0
44	1	0,00	0	4,8		110	0	0	105	1	4	0	0	0	0	0	0	0	0	0	0
88	1	0,00	0	4,7	24,98	101	0	0	94	7	4	1	0	0	0	0	1	0	0	0	2
93	0	0,00	0	5,6	23,73	96	0	0	151	17	4	1	1	0	0	0	0	0	0	0	0
86	0	0,00	0	4,6	29,97	103	0	0	100	2	4	1	0	0	0	0	0	0	0	0	0
47	0	0,00	0	4,8	32,95	109	0	0	126	1	4	1	0	0	0	0	0	0	0	0	0
69	0	0,00	0	5,1	34,02	110	0	0	110	4	4	1	0	0	0	0	0	0	0	0	0
77	0	0,00	0	5,0		102	0	0	84	2	4	1	1	0	1	0	0	0	0	0	0
78	1	0,00	0	5,3	23,67	90	0	0	71	1	4	0	0	0	0	0	1	0	0	0	0
81	0	0,00	0	5,6	29,59	90	0	0	152	21	4	1	0	0	0	0	0	0	0	0	0
82	0	0,00	0	4,8	31,22	106	0	0	115	5	4	0	1	0	0	0	0	0	0	0	0
75	1	0,00	0	4,8	31,25	96	0	0	99	3	4	0	1	0	0	0	1	0	0	0	0
77	1	0,00	0	5,5	31,83	102	0	0	150	23	4	1	1	1	1	1	0	0	0	0	0
70	0	0,00	0	5,0	26,50	89	0	0	133	3	4	1	1	0	0	0	0	0	0	0	0
49	1	0,00	0	4,4		100	0	0	103	15	4	0	0	0	0	0	0	1	0	0	0
75	1	0,00	0	5,5	32,87	104	0	0	142	4	4	1	1	0	0	0	0	0	0	0	0
67	1	0,00	0	4,9	28,40	91	0	0	120	6	4	0	0	0	0	0	0	0	0	0	0
78	0	0,00	0	5,2	25,39	83	0	0	209	4	4	1	1	0	0	0	0	0	0	0	0
80	1	0,00	0	5,1	25,25	94	0	0	127	23	4	1	1	0	0	0	0	0	0	0	0
53	1	0,00	0	5,0	25,16	97	0	0	110	9	4	1	0	0	0	0	0	0	0	0	0
68	1	0,00	0	5,6	25,06		0	0	102	3	4	0	0	0	0	0	1	0	0	0	0
82	0	0,00	0	4,9	31,22	100	0	0	94	3	4	1	0	0	0	0	0	0	0	0	0
83	0	0,00	0	4,8			0	0	92	9	4	1	1	0	1	0	0	0	0	0	1
68	1	0,00	0	4,8	25,31		0	0	102	20	4	1	0	0	0	0	1	1	0	0	0
70	0	0,00	0	4,8	28,52		0	0	123	4	4	1	0	0	0	0	0	0	0	0	0
78	1	0,00	0	5,2	19,61		0	0	139	3	4	1	1	0	0	0	0	0	0	0	0
83	0	0,00	0	5,3	29,59	92	0	0	106	14	4	0	0	0	0	0	0	0	0	0	0
72	1	0,00	0	4,4	27,55	93	0	0	116	12	4	1	0	0	0	1	0	0	0	0	2
79	1	0,00	0	4,8	32,27	111	0	0	122	2	4	1	1	0	1	0	1	0	0	0	0
78	1	0,00	0	5,2	23,89	103	0	0	106	6	4	1	0	0	0	0	0	0	0	0	0
76	0	0,00	0	5,2	26,06	100	0	0	115	9	4	1	1	0	0	0	0	0	0	0	0

72	0	0,00	0	4,9	29,72	99	0	0	95	2	4	1	0	0	0	0	0	0	0	0	0
82	1	0,00	0	5,3	18,73		0	0	91	2	4	1	0	0	0	1	0	0	0	0	2
72	1	0,00	0	5,3	24,57	72	0	0	153	10	4	1	0	0	1	0	1	1	0	0	1
60	1	0,00	0	4,9	25,33		0	0	97	6	4	0	1	0	0	0	1	1	0	0	1
56	0	0,00	0	5,4		99	0	0	215	16	4	0	0	0	0	0	0	0	0	0	0
89	0	0,00	0	5,3	20,81		0	0	90	6	4	1	1	1	0	0	0	0	0	0	0
80	1	0,00	0	5,2			0	0	227	16	4	1	0	0	0	0	0	0	0	0	0
80	0	0,00	0	5,3			0	0	139	13	4	1	0	0	0	0	0	0	0	0	2
76	0	0,00	0	5,3			0	0	97	2	4	1	0	0	0	1	0	0	0	0	3
64	0	0,00	0	5,4			0	0	102	16	4	1	1	0	0	0		1	0	0	0
70	0	0,00	0	5,5			0	0	103	7	4	1	1	0	0	0	0	0	0	0	0
56	1	0,00	0	5,4	27,55	98	0	0	185	19	4	0	0	0	0	0	0	1	0	0	2
42	1	0,00	0	5,2	28,06	105	0	0	104	5	4	0	0	0	0	0	0	0	0	0	0
89	0	0,00	0	5,6			0	0	105	12	4	1	1	0	0	1	0	0	0	0	3
89	0	0,00	0	5,6			0	0	106	7	4	1	0	0	0	0			0	0	3
66	0	0,00	0	5,6	29,78	91	0	0	103	17	4	0	1	0	0	0	0	0	0	0	0
50	0	0,00	0	5,2	23,12	86	0	0	78	10	4	0	0	0	0	0	1	1	0	0	0
83	1	0,00	0	5,3	27,92	104	0	0	95	2	4	1	0	0	1	0	0	0	0	0	0
51	1	0,00	0	5,6	29,63		0	0	105	8	4	0	1	0	0	0	0	1	0	0	0
74	0	0,00	0	5,4	28,44		0	0	134	1	4	0	0	0	0	0	0	0	0	0	0
81	1	0,00	0	5,2	23,99	94	0	0	184	1	4	1	1	0	1	0	0	0	0	0	0
82	1	0,00	0	5,2			0	0	94	10	4	1	0	0	0	0	0	0	0	0	0
80	0	0,00	0	5,6	28,44	102	0	0	83	5	4	1	0	0	0	0	0	0	0	0	1
75	0	0,00	0	5,5	29,24	105	0	0	136	16	4	0	0	0	0	0	0	0	0	0	0
50	1	0,00	0	5,6	24,49	82	0	0	109	4	4	0	0	0	0	0	1	1	0	0	0
56	1	0,00	0	5,4	25,85	89	0	0	115	3	4	0	1	0	0	0		1	0	0	3
72	0	0,00	0	5,3	24,46	90	0	0	101	2	4	0	1	0	0	0	0	0	0	0	0
37	1	0,00	0	5,0	25,44	###	0	0	100	2	4	0	0	0	0	0	0	0	0	0	0
85	0	0,00	0	5,4	20,81		0	0	109	10	4	1	0	0	0	0	0	0	0	0	3
70	0	0,00	0	5,2	22,04	76	0	0	100	23	4	1	1	0	0	0	0	0	0	0	0
70	0	0,00	0	5,0	36,33	114	0	0	130	6	4	1	0	0	0	0	0	0	0	0	0
32	1	0,00	0	5,2	24,44	87	0	0	98	7	4	0	0	0	0	0		1	0	0	0
75	1	0,00	0	5,4	24,80	100	0	0	90	7	4	1	1	0	0	1	0	0	0	0	3
65	0	0,00	0	5,6	17,26	82	0	0	90	2	4	1	0	0	0	0	1	1	0	0	0
77	0	0,00	0	5,5	38,21	118	0	0	110	12	4	1	0	0	0	0	0	0	0	0	0
32	1	0,00	0	5,1	23,55	70	0	0	100	0	4	0	0	0	0	0	0	0	0	0	0
86	0	0,00	0	5,6	27,12	98	0	0	104	5	4	1	1	0	0	0	0	0	0	0	3

91	0	0,00	0	5,3		102	0	0	82	8	4	1	0	0	0	0	0	0	0	0	3
85	0	0,00	0	5,6	20,81	92	0	0	98	6	4		1	0	0	0	0	0	0	0	0
68	1	0,00	0	5,0	25,38	90	0	0	116	5	4	0	0	0	0	0	1	0	0	0	0
83	0	0,00	0	5,2	24,98	90	0	0	121	5	4	1	1	0	0	0	0	0	0	0	2
89	1	0,00	0	4,7	30,45	118	0	0	104	3	4	1	0	0	0	0	0	0	0	0	3
83	0	0,00	0	5,6	19,81	87	0	0	98	14	4	1	1	0	1	0	0	0	0	0	0
43	0	0,00	0	4,8	21,36		0	0	105	6	4	0	0	0	0	0	0	1	0	0	0
81	1	1,00	1	5,7	27,04	99	0	0	140	3	4	1	0	0	1	1	0	0	0	0	2
69	1	1,00	1	5,5	25,56	98	0	0	112	6	4	0	0	0	0	0	1	1	0	0	0
56	1	1,00	1	5,4	29,76	102	0	0	106	1	4	1	1	0	0	0	1	1	0	0	0
82	0	1,00	1	5,4	24,14	98	0	0	116	1	4	1	0	0	0	1	0	0	0	0	0
78	1	1,00	1	5,4	24,57	85	0	0	126	5	4	1	0	0	0	0	0	1	0	0	2
82	1	1,00	1	5,7	21,37	100	0	0	99	5	4	1	0	0	0	1	0	0	0	0	0
82	1	1,00	1	5,7	23,88	78	0	0	114	7	4	0	0	0	0	1	1	0	0	0	2
85	1	1,00	1	6,1	24,09	102	0	0	86	19	4	1	0	0	1	0	1	0	0	0	0
83	0	1,00	1	5,3	30,48	106	0	0	115	7	4	1	0	0	0	0	0	0	0	0	2
86	0	1,00	1	5,7	25,00	104	0	0	104	4	4	0	0	0	0	0	0	0	0	0	0
84	0	1,00	1	5,3	24,61		0	0	116	4	4	0	0	0	0	0	0	0	0	0	0
63	1	1,00	1	5,4	21,26	90	0	0	90	15	4	1	0	0	0	0	0	1	0	0	2
92	0	1,00	1	5,5			0	0	134	8	4	0	0	0	0	0	0	0	0	0	3
94	0	1,00	1	5,6	22,22	75	0	0	104	14	4	1	0	0	0	0	0	0	0	0	2
78	1	1,00	1	5,7	28,52	99	0	0	116	2	4	1	0	0	0	0	0	0	0	0	0
84	0	1,00	1	5,9	29,38		0	0	115	7	4	1	0	0	0	0	0	0	0	0	3
85	1	1,00	1	5,8			0	0	120	19	4	1	0	1	0	0	0	0	0	0	0
59	1	1,00	1	6,1	25,71		0	0	103	16	4	0	0	0	0	0	0	1	0	0	3
71	0	1,00	1	6,4	24,22	103	0	0	134	8	4	0	0	0	0	0			0	0	0
88	1	1,00	1	6,4	24,22		0	0	80	3	4	1	1	0	1	0	0	0	0	0	1
81	0	1,00	1	5,7	29,14	101	0	0	103	6	4	1	1	0	0	0	0	0	0	0	0
43	0	1,00	1	5,7	28,89		0	0	103	3	4	1	0	0	0	0		0	0	0	0
80	1	1,00	1	5,7			0	0	88	3	4	1	0	0	0	0	0	0	0	0	0
81	1	1,00	1	5,8			0	0	153	0	4	1	0	0	0	0		0	0	0	2
91	0	1,00	1	5,8	20,20	81	0	0	140	5	4	0	0	0	0	0	0	0	0	0	0
86	1	1,00	1	5,9	30,10		0	0	132	9	4	0	0	0	0	0	0	0	0	0	0
73	0	1,00	1	5,8	26,17		0	0	120	3	4	1	0	0	0	0	0	0	0	0	2
73	0	1,00	1	5,7	31,22	118	0	0	144	12	4	1	0	0	0	0	0	0	0	0	0
83	0	1,00	1	5,8		93	0	0	109	2	4	0	1	0	0	0	0	0	0	0	2
87	1	1,00	1	6,3	26,67	100	0	0	115	14	4	1	0	0	0	0	0	0	0	0	1

73	1	1,00	1	6,0	39,06	125	0	0	91	2	4	1	1	0	0	0	0	0	0	0	1
87	0	1,00	1	6,0	27,26	98	0	0	96	4	4	0	1	0	0	0	0	0	0	0	1
75	0	1,00	1	5,9	29,14	108	0	0	104	24	4	1	0	0	0	0	1	0	0	0	0
75	0	1,00	1	6,0	23,73	98	0	0	142	5	4	1	1	0	0	0	0	0	0	0	0
61	1	1,00	1	6,0	21,67	81	0	0	86	2	4	1	0	0	0	1	0	1	0	0	0
56	1	1,00	1	6,4	25,06	108	0	0	126	7	4	1	0	0	0	0	0	0	0	0	0
84	1	1,00	1	6,4	23,56	92	0	0	90	14	4	1	0	0	0	0	1	0	0	0	0
75	0	1,00	1	6,1	27,25		0	0	144	13	4	0	0	0	1	0	0	0	0	0	0
87	0	1,00	1	6,0	21,88		0	0	90	8	4	1	0	0	0	0	0	0	0	0	0
80	0	1,00	1	6,3	22,27	90	0	0	107	8	4	1	0	0	0	0	0	0	0	0	0
74	1	1,00	1	5,9		50	0	0	96	14	4	0	0	0	0	0	1	0	0	0	1
90	1	1,00	1	5,8	27,34	91	0	0	112	14	4	1	0	0	1	0	0	0	0	0	0
83	1	1,00	1	6,3	24,51	104	0	0	111	21	4	1	1	0	1	0	1	0	0	0	2
88	1	1,00	1	5,7	20,45		0	0	95	7	4	1	1	0	1	0	0	0	0	0	0
62	1	1,00	1	6,1	17,30	84	0	0	100	5	4	0	1	0	0	0	1	1	0	0	0
46	0	1,00	1	5,9	20,55		0	0	128	12	4	0	1	0	0	0	0	1	0	0	0
61	1	1,00	1	6,0	29,07		0	0	89	8	4	1	1	0	1	0	1	1	0	0	0
71	1	1,00	1	5,9	30,86	108	0	0	98	3	4	0	0	0	0	1	0	0	0	0	0
46	0	1,00	1	6,0	26,57	80	0	0	102	15	4	0	0	0	0	0	0	1	0	0	0
41	1	1,00	1	5,9	19,49	66	0	0	93	15	4	0	0	0	0	0	0	1	0	0	0
79	1	1,00	1	5,7	26,22	103	0	0	117	13	4	0	0	0	0	0	1	0	0	0	0
86	0	1,00	1	6,1	36,44	110	0	0	136	10	4	1	0	0	0	0	0	0	0	0	3
79	0	1,00	1	5,9	29,34	116	0	0	112	5	4	1	0	0	0	0	0	0	0	0	0
71	0	1,00	1	5,9	31,74	107	0	0	104	15	4	1	0	0	0	0	0	0	0	0	0
87	0	1,00	1	5,9	22,66	73	0	0	93	21	4	0	0	0	0	0	0	0	0	0	3
88	0	1,00	1	6,2	26,48	62	0	0	191	7	4	1	0	0	0	1	0	0	0	0	3
76	1	1,00	1	5,8	33,22	107	0	0	140	19	4	1	0	0	0	0	1	1	0	0	1
80	1	1,00	1	6,1	25,59	108	0	0	122	13	4	0	1	1	0	0	0	1	0	0	2
86	0	1,00	1	5,9	28,91		0	0	121	1	4	1	1	0	0	0	0	0	0	0	2
67	1	1,00	1	5,7	27,68		0	0	152	7	4	0	1	1	1	0	1	0	0	0	1
85	1	1,00	1	6,0	27,18	120	0	0	97	4	4	1	1	0	1	1	0	0	0	0	2
73	0	1,00	1	5,7	28,91	110	0	0	102	1	4	1	0	0	1	0	0	0	0	0	1
74	1	1,00	1	6,4	39,09	185	0	0	97	2	4	1	0	0	1	1	0	0	0	0	1
50	1	1,00	1	5,7	31,67		0	0	101	23	4	0	0	0	0	0	1	1	0	0	0
84	1	1,00	1	6,1	24,69	95	0	0	120	6	4	1	1	0	0	0	0	0	0	0	3
62	1	1,00	1	5,8	40,75	122	0	0	175	3	4	1	1	0	0	0	0	1	0	0	3
65	1	1,00	1	5,7	21,71	109	0	0	123	5	4	1	0	0	0	0	0	0	0	0	2

49	0	1,00		1	6,4	27,68		0	0	112	2	4	1	0	0	0	0	1	0	0	0	0
76	0	1,00		1	5,8	22,04		0	0	151	15	4	1	0	0	0	0	0	0	0	0	0
87	0	1,00		1	6,1	18,82	83	0	0	234	12	4	1	0	1	0	0	0	0	0	0	1
68	1	4,00		2	6,5	22,41	109	1	1	148	2	4	0	0	0	0	0	0	0	0	0	0
56	1	2,00		2	10,5	21,83	80	1	1	454	19	4	1	1	1	1	0	0	1	0	0	0
77	1	2,00		2	6,6	23,67	107	1	1	187	1	4	1	1	0	1	1	0	0	0	0	1
91	0	2,00		2	6,6			1	1	118	8	4	1	0	0	1	1	0	0	0	0	2
86	0	2,00		2	7,1	28,13	121	1	1	155	16	4	0	0	0	0	0	0	0	0	0	1
73	1	2,00		2	8,2	20,24	100	1	1	209	1	4	1	1	0	0	0	0	1	0	0	2
77	0	2,00		2	7,4	21,48	89	1	1	130	4	4	0	0	0	0	0	0	0	0	0	0
75	1	2,00		2	6,5	30,42	105	1	1	121	1	4	1	0		0	0	0	0	0	0	2
82	1	2,00		2	6,5	27,10	96	1	1	144	7	4	1	0	0	0	0	0	0	0	0	0
63	1	2,00		2	7,7	25,39	110	1	1	187	5	4	0	0	0	0	0	1	1	0	0	1
86	0	0,00		2	6,8	22,52	78	1	1	121	3	4	1	1	0	0	0	0	0	0	0	3
83	1	0,00		2	6,8	29,07	126	1	1	118	6	4	1	0	0	0	0	1	0	0	0	0
No	N	No	0																			
